# Adherence and Psychosocial Well-Being During Pandemic-Associated Pre-deployment Quarantine

**DOI:** 10.3389/fpubh.2021.802180

**Published:** 2021-12-22

**Authors:** Antje Bühler, Gerd-Dieter Willmund

**Affiliations:** German Center for Military Mental Health, Military Hospital Berlin, Berlin, Germany

**Keywords:** quarantine, psychosocial impact, mental health, military, deployment, adherence—compliance, Covid-19

## Abstract

**Background:** With the purpose of preventing SARS-Cov-2 traveling with the troops, pre-deployment and post-deployment quarantine are mandatory for the German military. This study investigates which factors could be addressed in order to facilitate adherence and mental health during isolation.

**Method:** Six hundred three soldiers completed questionnaires at the beginning and at the end of pre-deployment quarantine: Mini-SCL (BSI), Perceived Social Support (FSozU-K22), Unit Cohesion, Military Quarantine Adherence Questionnaire (MQAQ), and quarantine-associated factors including informedness about Covid-19, perceived individual risk, benefit of quarantine, clarity of quarantine protocol, need of intimacy, social norms, stigma, practicality of the quarantine, financial disadvantages, boredom, and health promoting leadership.

**Results:** Using stepwise regression analyses, up to 57% of the quarantine adherence was explained by social norms, boredom, perceived benefit/effectiveness of the quarantine, clear communication of the quarantine protocol and perceived risk of an infection, with social norms explaining 43%. In respect to mental health (Mini-SCL) at the beginning of quarantine, only 15% is explained by being in a partnership, (un)fulfilled need for bonding/intimacy, perceived unit cohesion, and perceived social support. Up to 20 % of the variance in mental health at the end of quarantine is explained by accumulated days of isolation before pre-deployment quarantine, age, clear communication of the quarantine protocol, perceived social support, fulfilled need for bonding/intimacy and perceived stigma. Mental health and quarantine adherence did correlate significantly, but to a slight extent. No differences between the beginning and the end of pre-deployment quarantine were found for the overall group in respect to mental health, quarantine adherence, perceived social support and perceived unit cohesion, while their trajectories differed for different subgroups including age, gender, rank, and accumulated days of quarantine: With increasing accumulated days of isolation prior to pre-deployment quarantine, mental health declined over the course of quarantine, though to a small degree.

**Conclusion:** Findings suggest that addressing the norms of fellow soldiers and dependents alike could contribute to quarantine adherence in pre-deployment quarantine. Ongoing research should examine long-term effects on mental health, including these of accumulated days of quarantine, also taking into account post-deployment quarantine.

## Introduction

While around 17 million soldiers lost their lives in the First World War between 1914 and 1918 (1), at least 20-50 million people succumbed to the Spanish flu between 1918 and 1920 (2), according to individual estimates up to 100 million people (3) - with a world population of 1.8 billion. Nonetheless, it is reported (4) that in the Franco-Prussian War of 1870/71 and in World War I (WWI), for the first time, more soldiers were killed in combat than by infectious diseases, especially on the German side (4). The first-time low death-toll caused by infections is explained by sanitary and hygienic measures as well as the military ordering of vaccinations (4). However, troop movements also spread infectious diseases during WWI. Along with other factors, the spread of the “Spanish” flu is attributed to a transport of US troops (4, 5). Summing up, the armed forces have historically played a pioneering role in fighting epidemics and pandemics as well as in its spread and globalization.

During the current Coronavirus Disease 19 (Covid-19) pandemic, troops continue to be deployed worldwide, while the civilian population is called upon to stay at home, e g. by the Federal German Ministry of health (https://www.zusammengegencorona.de/wirbleibenzuhause/). Departments of Defense across the globe have issued force-specific health protection guidance for deployment and redeployment of individuals and units during the Covid-19 pandemic in 2020 (6, 7). Their purpose is to maintain the health and operational readiness of their own and allied troops as well as protecting the vulnerable population in the regions (countries) of deployment and their own population at home upon return of the troops. In addition to hygiene measures, soldiers have been ordered to quarantine 14 days prior to deployment and upon return home (7–9). Differing from quarantine and isolation measures for the civilian population, it is applied to soldiers with no previous contact with an infected person or a confirmed infection.

Containment of infections in the context of the current Covid-19 pandemic is only considered to be attainable, if people adhere to the quarantine or isolation rules. The effectiveness of early quarantine measures for reducing incidence and mortality is supported by a rapid review (10). Therefore, we are interested in quarantine adherence with this specific group and factors impacting on the adherence.

As the Parliamentary Commissioner of the German Bundeswehr (11) received several complaints concerning the hardships of pre-deployment quarantine, we are interested in finding out, if the deployment-related quarantine(s) have an impact on mental health and which quarantine-related factors potentially influence the mental health of the quarantined military personnel.

### Attitudes of Military Personnel Toward Post-deployment Quarantine and Mental Health

There is a dearth of research on military deployment-related quarantine. An exception is a cross-sectional study reporting on a 3-week collective post-deployment quarantine after a humanitarian logistic mission to Ebola-affected West-Africa in 2014 (12). The percentage of soldiers reporting significant symptoms of a mental health disorder (3.2%) at the end of a collective post-deployment quarantine (12), seems considerably low when comparing these to 2.4% pre-deployment and 5.8% during deployment on a humanitarian mission during the Ebola pandemic in Liberia in a different study (13) or to prevalence rates for deployment-related disorders with high combat-exposure in Iraq and Afghanistan, e.g., for PTSD 9-20% (14). However, reported sleeping problems, often a precursor for mental health problems, are considerably higher with 29.8% (12) as compared to 4.9% before and 12.4% during deployment to Liberia during the Ebola pandemic (13).

Factors associated with sleeping quality and a positive attitude toward the quarantine quality were perceived family support and health promoting leadership.

The low impact of post-deployment quarantine on mental health and positive attitude toward the quarantine seem to contrast with the results of many studies on mental health of civilians quarantined and their quarantine adherence (15, 16). Therefore, the factors singled out to influence civilians' quarantine adherence and civilians' mental health should be described in more detail, as at this point the results on post-deployment quarantine (12) cannot be generalized to the current Covid-19 pandemic for the following reasons: 1) the relative objective risk of infections changing between countries throughout the Covid-19 pandemic, often implying higher infection rates in the soldiers' home countries than during deployment, by contrast with a zero infection with Ebola domestically in 2014, 2) the additional pre-deployment quarantine, 3) the extent of physical isolation during individualized quarantine as opposed to collective confinement involving regular training (12), 4) the purpose of the foreign assignment, a potential combat mission vs. humanitarian support in an epidemic [Ebola, 2014], and 5) the voluntariness of the mission and related quarantine.

It can be assumed that these factors influence the attitudes and management of the quarantine situation as well as the protective factors of perceived social support, perceived military leadership and unit cohesion.

No relevant studies were found on how quarantine or isolation measures affect the protective factors of social support and unit cohesion for military personnel, when we used the search terms “unit cohesion and quarantine” and “unit cohesion and isolation” in the data bases ERIC, APA Psyndex, APA Psych Articles, PsycInfo, Medline, SCOPUS, and PubMed for articles up to 20/08/2021.

### Rates of Adherence With the Quarantine or Isolation Protocol and Factors Influencing Adherence

Internationally, adherence rates of the civilian population with the quarantine protocol vary between 0 and 92.8%, differing between people, groups of people and the occasion (16). These numbers are based on 14 studies between 2004 and 2018 in the context of various pandemics such as SARS, MERS and H1N1 influenza (16). To our knowledge, in Germany there are no statistics available on quarantine adherence of the German population in the current Covid-19 pandemic.

As to the questions which sociodemographic factors influence quarantine adherence positively, female gender (17, 18) and higher age (17, 19, 20) have been identified. Results for higher education are mixed (19, 21, 22). Mitigating factors could be the place of quarantine (19), individual or collective quarantine (22, 23).

While the knowledge about relevant sociodemographic factors indicates which groups should be addressed for improving adherence with the quarantine protocol, the question remains how to do so respective which psychological factors facilitate adherence.

Main factors found are knowledge respective clear information about the disease and quarantine procedure (24, 25), respective health literacy (23, 26), social norms in favor of quarantine adherence (or even social pressure) (16), perceived risk of the disease and perceived benefits of quarantine (16) as well as the practicability of the quarantine, including sufficient supply with food and necessary daily goods, and access to medical treatment, as well as financial security (16, 24, 27) and level of psychological distress (16, 23). Single studies indicate that the endorsement of ethical principles, including “citizen's duty,” “community mindedness,” and the “greater good” based on free will, could facilitate adherence (28–31). The threat of enforcement was found to have less an effect than the credibility of compliance-monitoring” (30).

### Impact of Quarantine on Mental Health

Rapid reviews (15, 32) and one meta-analysis (33) on the mental health impact of quarantine conclude that there is (“compelling”) “evidence for adverse mental health” effects, including anxiety and depressive disorders and stress-related disorders. However, most of the studies these reviews are based on are cross-sectional in nature. When comparing systematic reviews on pandemic lockdowns and home-confinement, we found that the three reviews which included cross-sectional studies and studies without control groups (34–36) found strong mental health effects, while a meta-analysis of exclusively longitudinal studies found small effects for mental health and concluded that most people were psychologically resilient to home confinement and lockdowns (37). To our knowledge, no meta-analyses are available analyzing the effect of quarantine on mental health including longitudinal or even prospective studies only. Therefore, we cannot rule out that effect sizes would decline when more methodological rigor is applied.

The systematic review (33) and the two rapid reviews (15, 32) describe sociodemographic and quarantine-related factors shaping the impact of the quarantine on mental health.

Single studies report sociodemographic factors constituting a higher vulnerability for certain groups, including younger age (38–40), lower levels of education, more severe financial consequences (38, 41–45), a history of previous mental illness and perceived physical health problems (33). However, exceptions are found for specific mental disorders, as alcohol abuse was found to be more prevalent with a higher economic status (46) and depressive disorders above the age of 55 (43).

Quarantine-related risk factors are previous exposure to infection and perceived risk of the infection, the duration of quarantine (15, 32, 33), dissatisfaction with the containment measures, in particular lacking provision with food, necessary supply and medical availability (15, 33), quarantine-related stigmatization (24, 47, 48) and lacking perceived social support (33, 45, 49, 50).

In the case of pre- and post-deployment quarantine, it is not only the quarantine which can impact on mental health but also the deployment itself resulting in an accumulation of stress factors.

### Military Deployment and Mental Health

Reported prevalence and incidence rates of deployment-related mental disorders vary between countries deploying soldiers and region of deployment, the methodologic rigor of the respective study and the procedure used for assigning a mental health disorder, based on a clinical interview vs. based on self-report questionnaires. For instance, prevalence of PTSD is higher for Iraq (12.9%; 95% CI 11.3% to 14.4%) than Afghanistan (7.1%; 95% CI 4.6% to 9.6%) (14). There is growing evidence that it is not deployment per-se affecting mental health, but combat during deployment (14, 51). This tendency is supported for the German Armed Forces: While the prevalence of mental disorders in Bundeswehr soldiers is generally lower than in a comparable civilian population (14.4 vs. 20%), the prevalence of panic disorders / agoraphobia and post-traumatic stress disorder is more common in soldiers with combat experience in foreign deployments than in the civilian population (52). The military-specific variant of social support “unit cohesion” has been shown in a large number of studies to be protective for mental health (53–56) and perceived social support in general (57). Here, again, we do not know how perceived social support in general and how unit cohesion in particular is affected by pre-deployment quarantine.

### Pre-deployment Quarantine During the Covid-19 Pandemic

Many of the factors, here described as being associated with quarantine adherence and quarantine-related mental health, were addressed in a standardized way during the pre-deployment quarantine of the German troops, thereby offering an (almost) quasi-experimental design: Soldiers are informed that the pre-deployment quarantine (“Isolierte Unterbringung”) is officially ordered on the instructions of the Ministry of Defense. They are briefed on the purpose of the quarantine and the quarantine protocol pre-quarantine and when in-processing in the quarantine facilities. Quarantinees are tested for the SARS-Cov-2 virus when in-processing into the quarantine and when out-processing. Concerning the practicality, quarantinees are provided with full-board in a hotel. They can order necessary daily goods and niceties online. A military organizational team in the hotel can be contacted 24/7. When necessary, medical care is provided for by a military GP. In addition, the phone number of a psychologist is offered. During pre-deployment quarantine, military personnel receive their regular salaries. In addition, they are compensated financially or by compensatory time-off for duty-related confinement 24/7. Violations of the quarantine-protocol are quite likely to be detected, investigated and result in disciplinary measures making them less likely. Prior to deployment, the health status of deploying soldiers must be screened.

Depending on assessed pandemic risk, immunization status, requirements by country of deployment, allied forces and international organizations, the required length of quarantined has been changed several times in 2021 (6, 58). Unlike the civilian population, quarantining and testing are mandatory for inoculated military personnel before (re)deployment.

Summing up, there is multiple evidence in favor and against pre-deployment quarantine affecting mental health and conditions of pre-deployment quarantine facilitating or obstructing quarantine adherence. So far, we also do not know how pre-deployment quarantine affects the health protective factors of perceived unit cohesion and perceived social support.

As a consequence of the current state of research, we are interested in the following questions:

1) Does pre-deployment quarantine affect the quarantinees' mental health, respectively does their mental health change over the course of pre-deployment quarantine?
2) Does quarantine adherence change over the course of quarantine?
3) Does pre-deployment quarantine affect perceived social support and perceived unit cohesion, respectively do they change over the course of pre-deployment quarantine?

Based on previous research (12, 15, 16, 33), we expect the following relationships between mental health and quarantine adherence on the one hand and presumed mental health and adherence facilitating factors on the other hand:

Hypothesis 1: Mental health predicts quarantine adherence.

Hypothesis 2: Mental health (Mini-SCL) is positively influenced by:

- Fewer accumulated days of quarantining,
- low perceived risk of infection,
- the perceived level of information about Covid-19,
- the perceived benefit of the quarantine,
- the perceived level of clarity regarding the quarantine protocol (purpose, duration, rules regarding isolation),
- low perceived costs including: a high perceived practicability of the quarantine (being well provided for during the quarantine) low stigmatization,
- compliance with social norms supporting quarantine adherence,
- a low level of boredom
- the level of perceived social support in general and military and quarantine-specific forms of perceived social support in particular:
  - unit cohesion and healthy leadership behavior and
  - fulfilled need for bonding/intimacy.

Hypothesis 3: Quarantine adherence is positively influenced by:

- fewer accumulated days of quarantining,
- a higher perceived risk of infection,
- the perceived level of information about Covid-19,
- health-promoting leadership behavior,
- the perceived benefit of the quarantine, foremost its preventative effectiveness,
- the perceived level of clarity regarding the quarantine protocol (purpose, duration, rules),
- low perceived costs including: a high perceived practicability of the quarantine (being well provided for during the quarantine)
- low stigmatization,
- compliance with social norms supporting quarantine adherence,
- a low level of boredom,
- fulfilled need for bonding/intimacy and
- the absence of financial disadvantages.

## Methodology

### Participants and Procedure

Data were collected during pre-deployment quarantine between February and July 2021. Administered informed consent had to be adapted to the quarantine protocol. PowerPoint presentations informed about the study as part of the inprocessing at the quarantine facility when soldiers were instructed about the quarantine protocol. In addition, soldiers interested in participating were informed by writing and provided phone numbers they could contact for further questions. Participants were enrolled in the study upon prior written consent.

### Measures

As this study forms part of a longitudinal design with up to five measurement points, the rationale for choosing assessment instruments was to reduce dropout by keeping completion time as short as possible while at the same time relying on reliable and valid measures, when available.

#### Mental Health

The Mini-SCL, the German version of the Brief Symptom Inventory, measures psychological distress (mental strain) during the last 7 days, thereby covering a relevant time period for the purpose of measuring short- and long-term effects of quarantine. The GSI-score of the Mini-SCL shows good convergent and discriminant validity and very high reliability (α≥0.90), while still being sensitive to change (59). It takes 1-2 min to complete the questionnaire. In addition, it provides norms for a broad range of age groups.

#### Military Quarantine Adherence Questionnaire (MQAQ)

As to our knowledge, no instrument was available measuring military quarantine adherence, we developed an eight-item scale, the “Military Quarantine Adherence Questionnaire (MQAQ).” The MQAQ is based one of the scales assessing medication adherence we considered most adequate, the *Medication Adherence Report Scale* of Horne (60) assessing the attitude toward the pre-deployment quarantine and quarantine protocol, struggling with the protocol on a daily basis and motivation to adhere on a daily basis. Based on the PCA with Varimax rotation, two components have been extracted, “attitude toward the quarantine protocol” and the “frequency of struggling with adhering to the protocol.” As only, the eight-item scale reached good internal consistency (α = 0.8), it is recommended to use one scale only (61).

#### Perceived Social Support

Perceived social support is measured by a short version (K-22) of the FSozU (62). While contentwise, the items allow to use subscales for different forms of perceived social support, including practical support, emotional support, social integration, trusted person and satisfaction with social support, it is recommended to use the short version K-22 as one scale (63–65). Reliability (Cronbach Alpha) is excellent with α = 0.91. Its criterion validity, including its convergent and discriminant validity (64), is good. Norms for clinical and non-clinical groups are available. Answering the questions of FSozU-K-22 takes 5 min.

#### Perceived Unit Cohesion, Health Promoting Leadership, Need for Intimacy/Bonding

Prior to the study, scales measuring perceived unit cohesion and perceived health promoting leadership have not been validated in German language. These two five-point Likert-scales have been validated with a separate sample (*n* = 148) (61). Health promoting leadership is a six-item scale capturing two components, (a) if the respective soldier believes his/her military leaders to be concerned about her/his physical and mental health (individual health promoting leadership) and (b) the degree to which military leaders focus on preventing infections with the Coronavirus (Covid-specific leadership). Perceived unit cohesion is rated on seven items describing the relationship with the soldier's military peers and military leaders. Exploratory PCA with varimax rotation yielded a three component structure, explaining 71.9% of the variance: (a) unit cohesion, (b) individual health promoting leadership, and (c) Covid-specific leadership. Internal consistencies range between good and excellent for the four subscales “perceived support by military supervisor” (α = 0.88), “perceived support by military peers” (α = 0.85), individual health promoting leadership (α = 0.90), and Covid-specific leadership (α = 0.87) and the two main scales, health promoting leadership (α = 0.89) and unit cohesion (α = 0.90). Criterion validity is supported by moderate positive correlations between the military specific scales “unit cohesion” and “health promoting leadership” (*r* = 0.36, *p* < 0.001, *n* = 138) and perceived social support (FSozU-K-22) correlating positively with unit cohesion (*r* = 0.28, *p* < 0.001, *n* = 138) and military-specific health promoting leadership (*r* = 0.19, *p* < 0.001, *n* = 138), though to a small degree (61).

#### Quarantine-Related Factors With Potential Impact on Mental Health and Adherence

In the absence of a validated scale capturing nine quarantine-related psychosocial factors potentially facilitating mental health and quarantine adherence, we assessed its psychometric properties with a separate sample of 152 soldiers quarantined. Subjecting 37 items to principal component analysis using nine components as a cut-off criterion and varimax rotation, seven of the nine conceptualized factors were extracted, explaining 59.23% of the variance: With one exception, the scales yielded satisfactory to good reliability (consistency). The factors respective scales are: (1) the four-item scale “Perceived knowledge about Covid” (α = 0.83), (2) the seven-item scale “Perceived benefit of the quarantine” (α = 0.83), (3) the five-item scale “Perceived risk of infection” (α = 0.74), perceived risk of infection (oneself, peers, relatives) (α = 0.85), (4) the six-item scale “Perceived practicality of the quarantine” (supply with food, medical care, information, loved ones is cared for) (α = 0.82), (5) the five-item scale “positive social norms toward the quarantine by relevant others” [short: social norms] (military peers and family) (α = 0.73), (6) the five-item scale “perceived stigmatization” (by fellow soldiers/peers) (α = 0.73), (7) the five-item scale “boredom” (α = 0.87), (8) the seven-item scale “Perceived clarity of communication concerning the quarantine protocol” (purpose, duration, rules relating to isolation) reaches acceptable consistency when the items are standardized (α = 0.75), and (9) the four-item scale “Fulfilled need for intimacy/bonding” (α = 0.59), including the aspects of intimacy, physical closeness and sexuality, and aspects of bonding, including contact to relevant others and holidays with relevant others.

The scale “clear communication of the quarantine protocol” reaches satisfactory reliability (α ≥ 0.7), when all items are standardized. Therefore, all items are z-standardized before calculating scale means.

#### Financial Disadvantage Due to the Quarantine

This aspect was captured by one item “Due to the quarantine, I am/my family is experiencing financial disadvantages (e.g., additional costs for child care, shortened deployment^1^, etc.).”

### Analysis

All analyses were carried out in SPSS 25. Required sample size was calculated with the help of GPower (66). When not available in SPSS 25, effect sizes and confidence intervals (CIs) were calculated manually with the help of the two websites, https://www.psychometrica.de/korrelation.html for correlations (67) and https://effect-size-calculator.herokuapp.com/ for partial eta squared (68) and omega squared (69).

### Sample

#### Required Sample Size

A sample size of 361 participants provides sufficient power for detecting changes between the beginning and the end of quarantine in respect to quarantine adherence, mental health, perceived social support, and unit cohesion (*F*-tests**—**ANOVA: Repeated measures, within factors, effect size *f* = 0.1, α err prob = 0.0125, power (1-β err prob) = 0.90, number of groups = 1, number of measurements = 2, corr among rep measures = 0.5) (66, 70).

Hypothesis 1, 2, and 3 were tested independently of each other. As these hypotheses are directed, the initial error probability is α = 0.1. With the objective of testing hypotheses 1, three correlations were calculated, and for testing hypothesis 2 and 3, three linear multiple stepwise regressions were carried out, respectively. The error probability was adjusted accordingly at α = 0.03. The a priori computed required sample size is 352 (*F*-tests**—**Linear multiple regression: Fixed model, *R*^2^ deviation from zero, effect size *f*^2^ = 0.10, α err prob = 0.03, power (1-β err prob) = 0.90) (66).

#### The Sample and Accounting for Potential Bias

Six hundred three soldiers in pre-deployment quarantine participated in the study. Due to missing sociodemographic data, the sample size could be reduced to *n* = 470. The minimal sample size still constituted over-recruitment: For most of the sociodemographic variables, missing data remained less than *n* = 10 (1.69%) for each of the variables included and was not correlated with any of the variables resulting in no potential bias. The largest percentage of data exclusion can be attributed to lacking information on accumulated days of quarantine, 7.26% (*n* = 43).

#### Sociodemographics

Participants were between 18 and 64 years old with a mean age of 35 years (*SD* = 8.5 years). 88.2% were male and 10.6 female. 12.6% had a lower rank (enlisted personnel/private/corporal), 51.7% a middle rank (non-commissioned officers), and 31.7% had a higher rank (commissioned officer). 55.1% served as regular service members (under a limited contract) and 36.2% served as professional servicemembers (under an unlimited contract). Temporarily enlisted soldiers constituted 0.9% and reserve soldiers 4%.

78.8% were in a partnership and 19.4% were single. Soldiers with and without children (49.1%, respectively) were fairly distributed. 2.2% of all participants in the study were single caretakers, making up 4.4% of all caretakers. During the pandemic, a fourth of all caretakers (25.2%) had to leave their kids with the pandemic-specific emergency care.

Number of previous deployments ranged between 0 and 40 times (resulting in 1,500 days in theater) with a median of 2.38. 28.9% have not been deployed before, 21.6% once, 14.4% twice, 9.8% three times and 7.3% at least four times. The median for cumulative days of deployment was 217.5 days; the maximum was 1,680 days in theater.

67.5% reported having been ordered to quarantine before the pre-deployment quarantine: 48.6% once, 13.4% twice and 3.3% three times and 1.3% at least four times. The maximum number of previous days in quarantine reported was 307 days with a median of 9.32 days in a previous quarantine. 1.4% reported having been quarantined more than 50 days prior to the pre-deployment quarantine. Numbers of days in quarantine does not only refer to pre-deployment quarantine, but refers to all forms of quarantining prior to the pre-deployment quarantine. Though, a sum of 307 days in quarantine raises questions. Excluding data might cause biased results as well as including an extreme potentially unreliable case. Therefore, we carried out the statistics with all cases included and controlled for a potential bias by carrying out the same calculation after this case had been eliminated. Only deviating results will be reported for the sake of readability.

## Results

### Changes of Mental Health, Quarantine Adherence, Perceived Social Support, and Unit Cohesion Over the Course of Pre-deployment Quarantine

Four one-way repeated measures ANOVAs were conducted with the dependent variables mental health (MINI-SCL), quarantine adherence, perceived social support, and perceived unit cohesion. The following innersubject factors were entered: age, gender, partnership, number of children, single caretaker, children in pandemic emergency care, rank, accumulated days of deployment, and accumulated days of quarantine at the beginning of quarantine.

Using Pillai's trace, the ANOVAs yielded no significant differences between the beginning and the end of quarantine for quarantine adherence, *V* = 0.000, *F*_(1, 466)_ = 0.045, *p* = 0.832, η^2^ = 0.000, LL CI_97, 5%_ = 0, UL CI_97.5%_ = 0.011, ω^2^ = 0, mental health (Mini-SCL), *V* = 0.000, *F*_(1, 462)_ = 0.016, *p* = 0.900, η^2^ = 0.000, LL CI_97.5%_ = 0, UL CI_97.5%_ = 0.008, ω^2^ = 0, perceived social support, *V* = 0.008, *F*_(1, 462)_ = 3.796, *p* = 0.052, η^2^ = 0.008, LL CI_97.5%_ = 0, UL CI_97.5%_ = 0.037, ω^2^ = 0.006, and perceived unit cohesion, *V* = 0.000, *F*_(1, 447)_ = 0.205, *p* = 0.651, η^2^ = 0.000, LL CI_97.5%_ = 0, UL CI_97.5%_ = 0.016, ω^2^ = 0.

**Figure 1:**
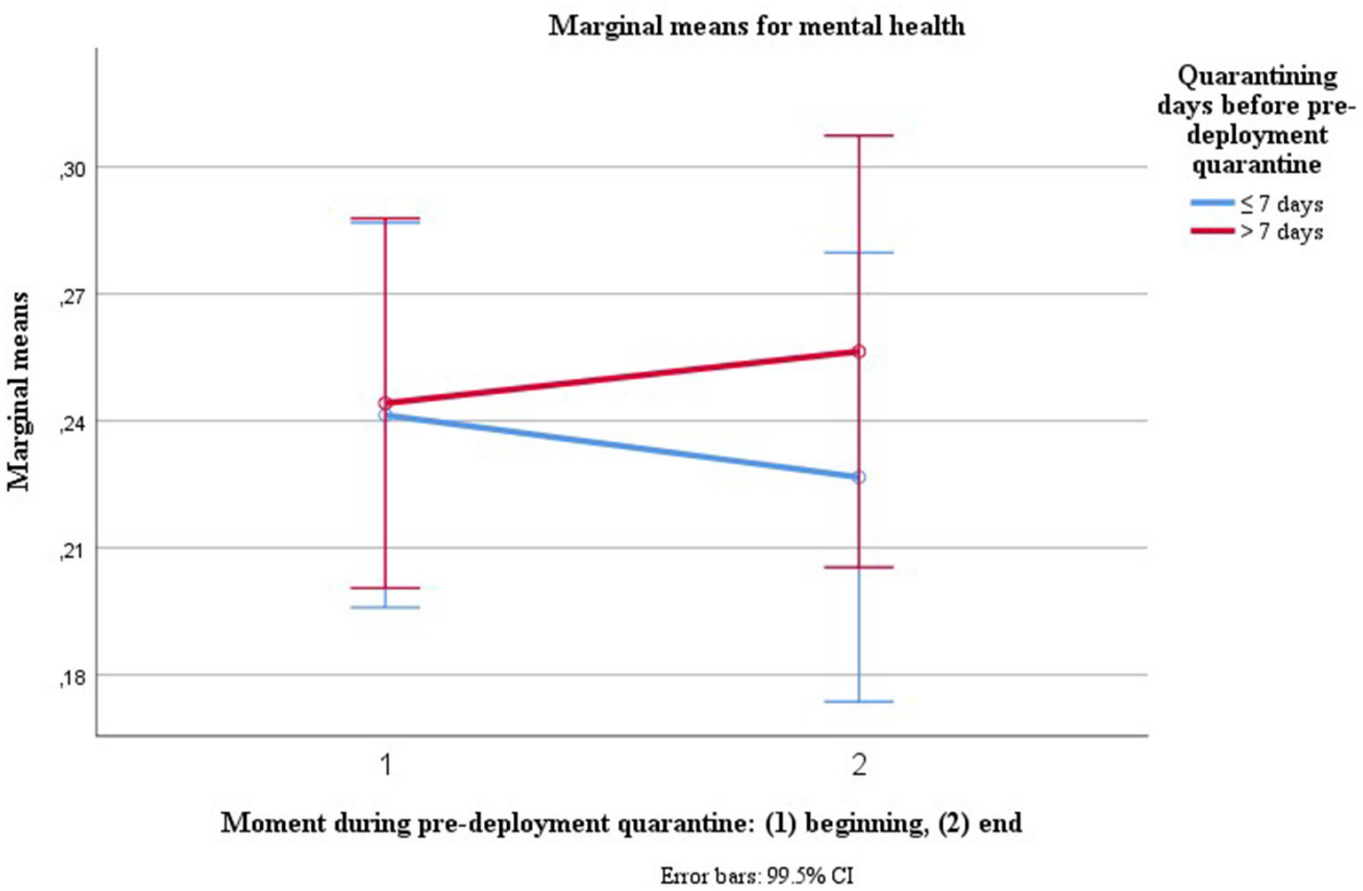
Trajectories for mental health dependent on previous quarantining.

“Accumulated days of quarantining prior to pre-deployment quarantine” was the only covariate (sociodemographic variable) influencing mental health over the course of pre-deployment quarantine [*V* = 0.02, *F*_(1, 462)_ = 8.313, *p* = 0.004, η^2^ = 0.018, LL CI_97.5%_ = 0.001, UL CI_97.5%_ = 0.054, ω^2^ = 0.016], predicting a slight decline of mental health over the course of quarantine (see Figure 1). After excluding the extreme case with reportedly 307 days in quarantine, the effect became more pronounced [*V* = 0.04, *F*_(1, 461)_ = 19.391, *p* = 0.000, η^2^ = 0.04, LL CI_97.5%_ = 0.010, UL CI_97.5%_ = 0.088, ω^2^ = 0.038].

As the figure for plotting mental health curves depending on accumulated days of quarantine and moment during quarantine is confusing, we transformed accumulated days of prior quarantining into a dichotomous variable, using cut-offs of typical quarantining periods, here 1–4 weeks of prior quarantining. The effect is stable for all cut-offs. Here, Figure 1 is illustrating the effect using a cut-off of 1 weeks of prior quarantining or isolation before pre-deployment quarantine.

**Figure 2:**
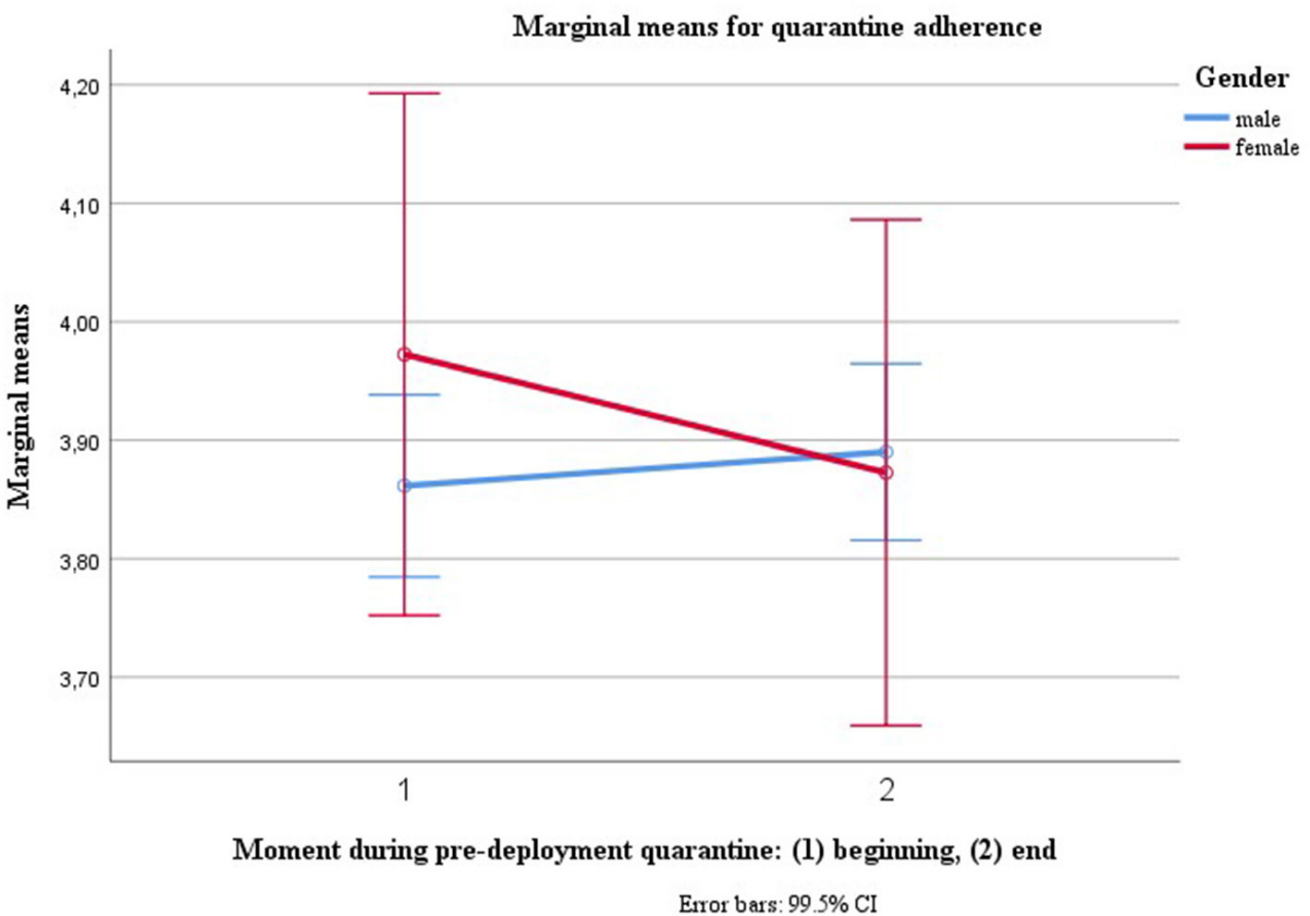
Trajectories for quarantine adherence for male and female soldiers.

Gender [*V* = 0.016, *F*_(1, 466)_ = 7.765, *p* = 0.006, η^2^ = 0.016, LL CI_97.5%_ = 0.001, UL CI_97.5%_ = 0.051, ω^2^ = 0.014] was the only innersubject factor to predict changes in quarantine adherence, predicting a decline in adherence over time and adherence remaining constant for male gender (see Figure 2).

As we understand gender as a proxy variable, we explored *post-hoc* which psychosocial factors, assessed at the beginning of quarantine, could explain the decline in adherence for female gender. The only variable correlating negatively with quarantine adherence (*r* = −0.14, *n* = 590, *p* < 0.000, t2: *R* = −0.23, *p* < 0.001, *n* = 590) and correlating positively with gender, though non-significantly (t1: R.043, *p* = 1.1, *n* = 587, t2: *r* = 0.07, *p* = 0.02, *n* = 584), was mental health symptoms (Mini-SCL). When adding the variable “mental health symptoms at the beginning of the quarantine” as a between-subject factor into the Repeated Measures ANOVA [*V* = 0.68, *F*_(1, 204)_ = 1.55, *p* = 0.001, η^2^ = 0.68 LL CI_97.5%_ = 0.08, UL CI_97.5%_ = 0.37, ω^2^ = 0.24], then the gender effect disappeared [*V* = 0.011, *F*_(1, 204)_ = 2.17, *p* = 0.146, η^2^ = 0.01 LL CI_97.5%_ = 0.00, UL CI_97.5%_ = 0.06, ω^2^ = 0.01].

**Figure 3:**
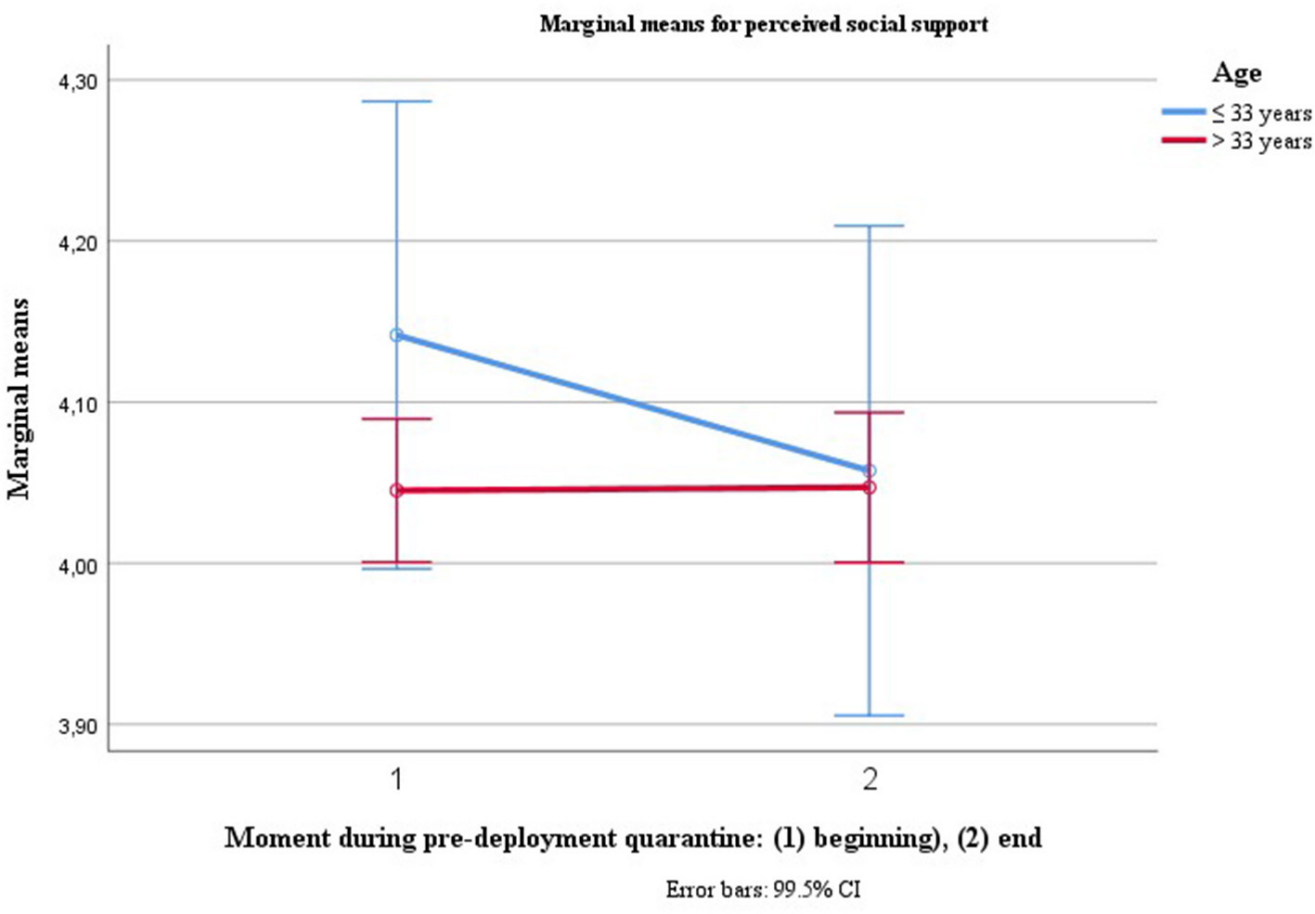
Trajectories for perceived social support depending on age.

For perceived social support, two of the inner subject factors, age [*V* = 0.018, *F*_(1, 462)_ = 8.534, *p* = 0.004, η^2^ = 0.018, LL CI_97.5%_ = 0.001, UL CI_97.5%_ = 0.054, ω^2^ = 0.016] and accumulated days of quarantines at the beginning of quarantine [*V* = 0.036, *F*_(1, 462)_ = 17.381, *p* = 0.000, η^2^ = 0.036, LL CI_97.5%_ = 0.008, UL CI_97.5%_ = 0.082, ω^2^ = 0.034], predicted a decrease in perceived social support over the course of pre-deployment quarantine (see Figure 3). When the extreme case with reportedly 307 days in quarantine is eliminated, the tendency of accumulated days of quarantine pre-quarantine remains, but is not significant anymore with Bonferroni-correction [*V* = 0.012, *F*_(1, 461)_ = 5.819, *p* = 0.016, η^2^ = 0.012, LL CI_97.5%_ = 0, UL CI_97.5%_ = 045, ω^2^ = 0.010].

**Figure 4:**
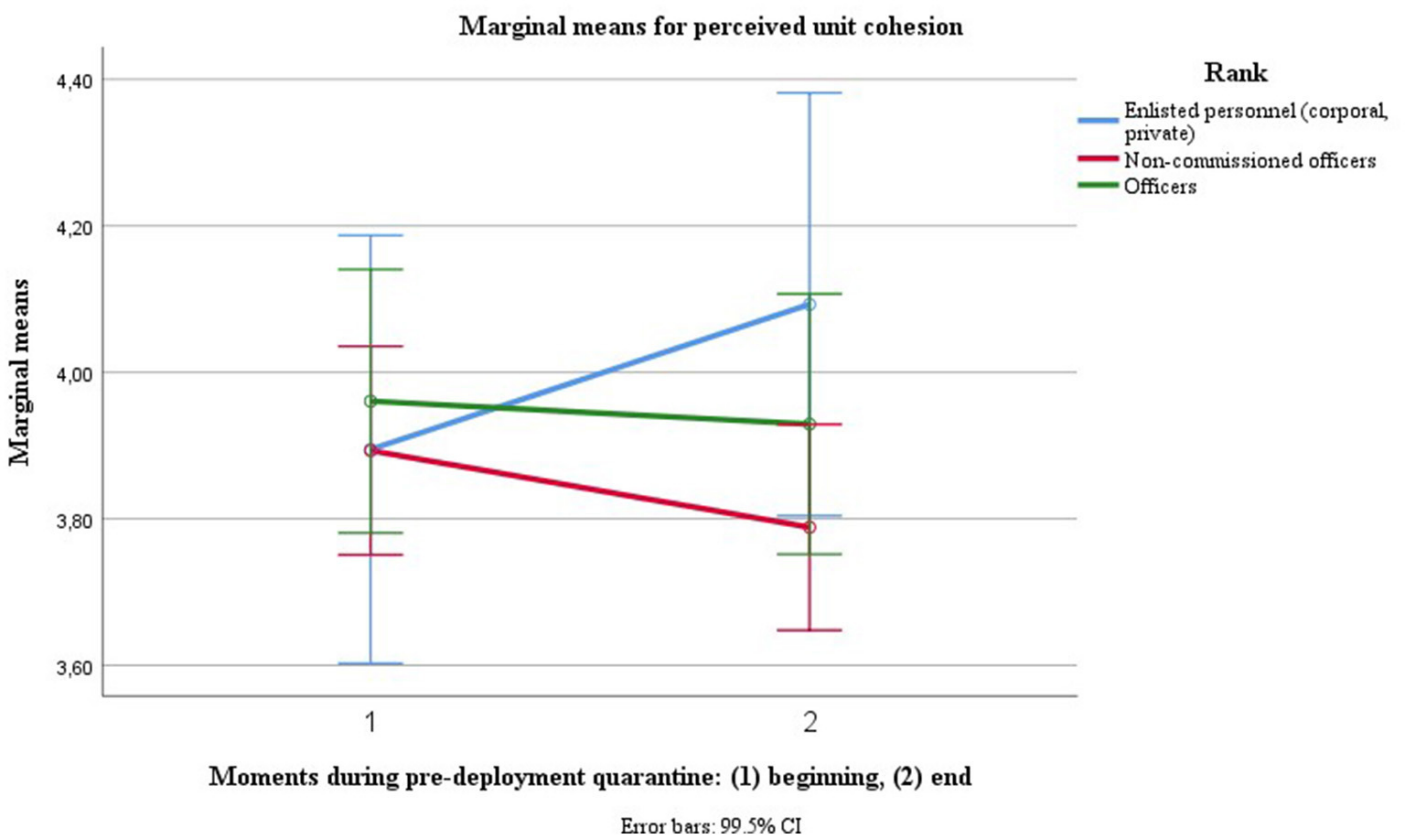
Trajectories for perceived unit cohesion dependent on rank.

The only innersubject factor predicting change in perceived unit cohesion over the course of quarantine was rank [*V* = 0.020, *F*_(1, 447)_ = 9.262, *p* = 0.002, η^2^ = 0.020, LL CI_97.5%_ = 0.001, UL CI_97.5%_ = 0.058, ω^2^ = 0.018], with lower ranks' (enlisted personnel's) adherence increasing over time, middle ranks' (non-commissioned officers') decreasing and higher ranks' (commissioned officers') remaining constant (see Figure 4).

### Predicting Mental Health and Adherence During Pre-deployment Quarantine

#### Hypothesis 1: Mental Health Predicts Quarantine Adherence

Mental health at the beginning of quarantine (Mini-SCL) and adherence at the end of quarantine are significantly related (*r* = −0.16, *p* < 0.001, *n* = 586, Fisher's *z* = −0.16, LL CI_90%_ = −0.23, UL CI_90%_ = −0.09). Mental health and quarantine adherence at the end of quarantine are significantly related (*r* = −0.30, *p* < 0.001, *n* = 588, Fisher's *z* = −0.31, LL CI_90%_ = −0.36, UL CI_90%_ = −0.24).

#### Hypothesis 2 and 3

Multiple stepwise regressions were calculated in order to predict mental health and quarantine adherence at different moments during the pre-deployment quarantine based on nine sociodemographic variables and 10 respective psychosocial variables. The nine sociodemographic predictors were age, gender, family status, number of children, single caretaker, children in pandemic-specific emergency care, accumulated days on deployment, and accumulated days quarantined when entering the pre-deployment quarantine. The identical 10 psychosocial predictors included health promoting leadership, feeling well-informed about Covid, perceived risk of a Covid infection, clarity of the quarantine protocol, perceived benefit/effectiveness of the quarantine, social norms supporting the quarantine, fulfilled need for bonding/intimacy, boredom, and financial disadvantage due to the quarantine. When predicting mental health perceived social support and perceived unit cohesion were added to the predictors.

Potential collinearity due to intercorrelations between the predictors (see Supplementary Material) were addressed by carrying out stepwise regression analysis. The mean of all psychosocial variables was based on z-standardized items. When assumptions were violated, including the normal distribution of the residuals or outliers in casewise diagnostics (> *SD* = 3), the robustness of the model was tested by bootstrapping, when entering the predictors identified in stepwise regression analysis. Tables with correlations between sociodemographic variables, quarantine-related predictors and dependent variables can be found are documented in the Supplementary Material as well as potential changes in quarantine adherence and its predictors over the months.

##### Mental Health During the Course of Quarantine

*Mental Health at the Beginning of Quarantine*. A significant regression equation was found predicting mental health at the beginning of the quarantine, respective symptom severity (Mini_SCL) [*F*_(4,526)_ = 25.50, *p* < 0.001], with family status, fulfilled need for bonding/intimacy, unit cohesion and perceived social support explaining 15% of the variance (*R* = 0.40, *R*^2^ = 0.16, corrected *R*^2^ = 0.15, LL CI_94%_ = 0.11, LL CI_94%_ = 0.21, ω^2^ = 0.16), with fulfilled need for bonding/intimacy [Δ*R*^2^ = 0.09, *F*_(1,529)_ = 49.97, *p* < 0.001, LL CI_99%_ = 0.04, LL CI_99%_ = 0.15, ω^2^ = 0.08] explaining most of the variance.

Testing the robustness of the model by bootstrapping, the predictors identified “family status, unit cohesion, perceived social support, and fulfilled need for bonding/intimacy” were entered in regression analysis. The results of stepwise regression were supported by using bootstrapping in regression analysis [*F*_(4,564)_ = 26.074, *p* < 0.001, *R*^2^ = 0.16, corrected *R*^2^ = 0.15, LL CI_94%_ = 0.10, UL CI_94%_ = 0.20, ω^2^ = 0.15], again with fulfilled need for bonding/intimacy [Δ*R*^2^ = 0.08, *F*_(1,567)_ = 45.71, *p* < 0.001, LL CI_99%_ = 0.10, UL CI_99%_ = 0.20, ω^2^ = 0.07] being the strongest predictor (see Table 1).

**Table 1:** Explaining mental health at the beginning of pre-deployment quarantine.

		**B**	**SE**	**Beta**	**T**	***p***	**LL CI_**95.5%**_**	**UL CI_**95.5%**_**	***r* (zero order)**
1	(Constant)	0.014	0.024		0.594	0.553	−0.034	0.063	
	zt1_Need for bonding	−0.245	0.036	−0.273	−6.761	0.000	−0.318	−0.172	−0.273
2	(Constant)	0.014	0.024		0.603	0.547	−0.033	0.062	
	zt1_Need for bonding	−0.224	0.036	−0.250	−6.284	0.000	−0.296	−0.153	−0.273
	zt1_Unit cohesion	−0.159	0.031	−0.205	−5.165	0.000	−0.220	−0.097	−0.234
3	(Constant)	0.189	0.053		3.557	0.000	0.082	0.296	
	zt1_Need for bonding	−0.236	0.035	−0.263	−6.657	0.000	−0.307	−0.165	−0.273
	zt1_Unit cohesion	−0.170	0.031	−0.219	−5.553	0.000	−0.231	−0.108	−0.234
	Partnership	−0.218	0.060	−0.145	−3.664	0.000	−0.338	−0.099	−0.094
4	(Constant)	0.164	0.053		3.090	0.002	0.057	0.271	
	zt1_Need for bonding	−0.245	0.035	−0.273	−6.957	0.000	−0.315	−0.174	−0.273
	zt1_Unit cohesion	−0.140	0.031	−0.182	−4.488	0.000	−0.203	−0.078	−0.234
	Partnership	−0.187	0.060	−0.124	−3.140	0.002	−0.307	−0.067	−0.094
	zt1_Social support	−0.177	0.049	−0.145	−3.592	0.000	−0.276	−0.078	−0.189

*Linear model of predictors with 95.5% bias corrected and accelerated confidence intervals (BCa CI). CI and standard errors (SE) based on 2,000 bootstrap samples. R^2^ = 0.08 for Step 1, ΔR^2^ = 0.04 for Step 2, ΔR^2^ = 0.02 for Step 3, ΔR^2^ = 0.02 for Step 4, (ps <0.001)*.

*Predicting Mental Health at the End of Quarantine*. A significant regression equation was found predicting mental health at the end of the quarantine, respective symptom severity (Mini_SCL) [*F*_(5, 525)_ = 17.42, *p* < 0.001] by predictors assessed at the beginning of quarantine. The predictors explain 14% of the variance (*R* = 0.40, *R*^2^ = 0.16, corrected *R*^2^ = 0.14, LL CI_94%_ = 0.09, UL CI_94%_ = 0.19, ω^2^ = 0.14): age, accumulated days of quarantine before pre-deployment quarantine, fulfilled need for bonding/intimacy, perceived social support and boredom. Fulfilled need for bonding/intimacy [Δ*R*^2^ = 0.05, *F*_(1,529)_ = 28.84, *p* < 0.001, LL CI_99%_ = 0.01, UL CI_99%_ = 0.11, ω^2^ = 0.15] and perceived social support [Δ*R*^2^ = 0.04, *F*_(1,528)_ = 24.27, *p* < 0.001, LL CI_99%_ = 0.01, UL CI_99%_ = 0.10, ω^2^ = 0.04] were the strongest predictors. Testing the robustness of the model by bootstrapping, the predictors identified in exploratory stepwise regression analysis were entered in regression analysis. The results of stepwise regression were supported by using bootstrapping in regression analysis [*F*_(5, 542)_ = 17.98, *p* < 0.001, *R* = 0.38, *R*^2^ = 0.14, corrected *R*^2^ = 0.13, LL CI_94%_ = 0.09, UL CI_94%_ = 0.19, ω^2^ = 0.13], with perceived social support [Δ*R*^2^ = 0.05, *F*_(1,544)_ = 26.30, *p* < 0.001, LL CI_99%_ = 0.01, UL CI_99%_ = 0.10, ω^2^ = 0.04] and fulfilled need for bonding/intimacy [Δ*R*^2^ = 0.05, *F*_(1,543)_ = 29.95, *p* < 0.001, LL CI_99%_ = 0.01, UL CI_99%_ = 0.11, ω^2^ = 0.05] being the strongest predictors (see Table 2).

**Table 2:** Predicting mental health at the end of quarantine.

		**B**	**SE**	**Beta**	**T**	***p***	**LL CI_**95.5%**_**	**UL CI_**95.5%**_**	***r* (zero order)**
1	(Constant)	−0.044	0.029		−1.498	0.135	−0.103	0.015	
	Quarantining days before pre-deployment quarantine	0.005	0.001	0.137	3.221	0.001	0.002	0.008	0.137
2	(Constant)	0.271	0.111		2.436	0.015	0.047	0.494	
	Quarantining days before pre-deployment quarantine	0.005	0.001	0.140	3.318	0.001	0.002	0.008	0.137
	Age	−0.009	0.003	−0.124	−2.935	0.003	−0.015	−0.003	−0.120
3	(Constant)	0.221	0.109		2.023	0.044	0.001	0.440	
	Quarantining days before pre-deployment quarantine	0.005	0.001	0.131	3.173	0.002	0.002	0.007	0.137
	Age	−0.007	0.003	−0.103	−2.487	0.013	−0.013	−0.001	−0.120
	zt1_need for bonding	−0.195	0.038	−0.212	−5.128	0.000	−0.271	−0.118	−0.227
4	(Constant)	0.287	0.107		2.678	0.008	0.072	0.502	
	Quarantining days before pre-deployment quarantine	0.005	0.001	0.134	3.346	0.001	0.002	0.007	0.137
	Age	−0.009	0.003	−0.129	−3.183	0.002	−0.015	−0.003	−0.120
	zt1_need for bonding	−0.203	0.037	−0.221	−5.475	0.000	−0.277	−0.128	−0.227
	zt1_social support	−0.276	0.050	−0.221	−5.473	0.000	−0.377	−0.175	−0.192
5	(Constant)	0.527	0.131		4.028	0.000	0.264	0.789	
	Quarantining days before pre-deployment quarantine	0.005	0.001	0.139	3.499	0.001	0.002	0.008	0.137
	Age	−0.009	0.003	−0.120	−2.977	0.003	−0.015	−0.003	−0.120
	zt1_need for bonding	−0.166	0.038	−0.182	−4.327	0.000	−0.244	−0.089	−0.227
	zt1_social support	−0.272	0.050	−0.218	−5.438	0.000	−0.373	−0.172	−0.192
	zt1_boredom	−0.079	0.025	−0.132	−3.144	0.002	−0.130	−0.029	−0.194

*Linear model of predictors, with 95.5% BCa CI. CI and SE based on 2,000 bootstrap samples. R^2^ = 0.02 for Step 1, ΔR^2^ = 0.02 for Step 2, ΔR^2^ = 0.05 for Step 3, ΔR^2^ = 0.05 for Step 4, R^2^ = 0.02 for Step 5, (ps <0.001)*.

*Explaining Mental Health at the End of Quarantine*. A significant regression equation was found predicting mental health at the end of the quarantine, respective symptom severity (Mini_SCL) [*F*_(7, 523)_ = 20.72, *p* < 0.001] by predictors assessed at the end of quarantine. The predictors explain 20% of the variance (*R* = 0.46, *R*^2^ = 0.21, corrected *R*^2^ = 0.20, LL CI_94%_ = 0.15, UL CI_94%_ = 0.26, ω^2^ = 0.21): accumulated days of quarantine before pre-deployment quarantine, age, clear communication of the quarantine protocol, perceived social support, fulfilled need for bonding/intimacy and perceived stigma, with clear communication of the quarantine protocol [Δ*R*^2^ = 0.08, *F*_(1,527)_ = 47.31, *p* < 0.001, LL CI_99%_ = 0.03, UL CI_99%_ = 0.15, ω^2^ = 0.08] being the strongest predictor.

Testing the robustness of the model by bootstrapping, the predictors identified in exploratory stepwise regression analysis were entered in regression analysis. The results of stepwise regression were supported by using bootstrapping in regression analysis [*F*_(6, 539)_ = 24.26, *p* < 0.001, *R* = 0.46, *R*^2^ = 0.21, corrected *R*^2^ = 0.20, LL CI_94%_ = 0.15, UL CI_94%_ = 0.26, ω^2^ = 0.20], with clear communication of the quarantine protocol [Δ*R*^2^ = 0.08, *F*_(1,542)_ = 48.66, *p* < 0.001, LL CI_99%_ = 0.03, UL CI_99%_ = 0.15, ω^2^ = 0.08] and perceived social support being the strongest predictors [Δ*R*^2^ = 0.05, *F*_(1,541)_ = 29.72, *p* < 0.001, LL CI_99%_ = 0.01, LL CI_99%_ = 0.11, ω^2^ = 0.05] (see Table 3).

**Table 3:** Explaining mental health at the end of quarantine.

		**B**	**SE**	**Beta**	**T**	***p***	**LL CI_**95.5%**_**	**UL CI_**95.5%**_**	***r* (zero order)**
1	(Constant)	−0.044	0.029		−1.495	0.135	−0.103	0.015	
	Quarantining days before pre-deployment quarantine	0.005	0.001	0.137	3.215	0.001	0.002	0.008	0.137
2	(Constant)	0.271	0.111		2.431	0.015	0.047	0.495	
	Quarantining days before pre-deployment quarantine	0.005	0.001	0.140	3.311	0.001	0.002	0.008	0.137
	Age	−0.009	0.003	−0.124	−2.930	0.004	−0.015	−0.003	−0.120
3	(Constant)	0.222	0.107		2.070	0.039	0.007	0.437	
	Quarantining days before pre-deployment quarantine	0.004	0.001	0.121	2.991	0.003	0.001	0.007	0.137
	Age	−0.007	0.003	−0.102	−2.514	0.012	−0.013	−0.001	−0.120
	zt2_clear quarantine protocol	−0.277	0.040	−0.283	−6.975	0.000	−0.357	−0.197	−0.299
4	(Constant)	0.253	0.104		2.420	0.016	0.043	0.463	
	Quarantining days before pre-deployment quarantine	0.004	0.001	0.106	2.671	0.008	0.001	0.006	0.137
	Age	−0.008	0.003	−0.112	−2.836	0.005	−0.014	−0.002	−0.120
	zt2_clear quarantine protocol	−0.252	0.039	−0.257	−6.444	0.000	−0.330	−0.173	−0.299
	zt2_social support	−0.250	0.046	−0.217	−5.452	0.000	−0.342	−0.158	−0.253
5	(Constant)	0.221	0.103		2.151	0.032	0.015	0.427	
	Quarantining days before pre-deployment quarantine	0.004	0.001	0.102	2.627	0.009	0.001	0.006	0.137
	Age	−0.007	0.003	−0.099	−2.552	0.011	−0.013	−0.002	−0.120
	zt2_clear quarantine protocol	−0.212	0.039	−0.217	−5.422	0.000	−0.290	−0.133	−0.299
	zt2_social support	−0.254	0.045	−0.221	−5.669	0.000	−0.345	−0.164	−0.253
	zt2_Need for bonding	−0.175	0.036	−0.194	−4.906	0.000	−0.247	−0.103	−0.252
6	(Constant)	0.247	0.102		2.423	0.016	0.042	0.451	
	Quarantining days before pre-deployment quarantine	0.003	0.001	0.093	2.424	0.016	0.001	0.006	0.137
	Age	−0.008	0.003	−0.108	−2.809	0.005	−0.013	−0.002	−0.120
	zt2_clear quarantine protocol	−0.178	0.040	−0.182	−4.454	0.000	−0.258	−0.098	−0.299
	zt2_social support	−0.225	0.045	−0.196	−4.972	0.000			
	zt2_need for bonding	−0.150	0.036	−0.166	−4.152	0.000			
	zt2_stigma	−0.122	0.036	−0.143	−3.422	0.001			

*Linear model of predictors with 95.5% BCa CI. CI and SE based on 2,000 bootstrap samples. R^2^ = 0.02 for Step 1, ΔR^2^ = 0.02 for Step 2, ΔR^2^ = 0.08 for Step 3, ΔR^2^ = 0.05 for Step 4, ΔR^2^ = 0.04 for Step 5, ΔR^2^ = 0.02 for Step 6, (ps <0.001)*.

##### Adherence During the Course of Quarantine

*Quarantine Adherence at the Beginning of Quarantine*. A significant regression equation was found predicting quarantine at the beginning of the quarantine [*F*_(5, 525)_ = 89.87, *p* < 0.001]. The variables social norms, perceived benefit/effectiveness of pre-deployment quarantine, boredom, perceived risk of infection with SARS-CoV2 and clear communication of the quarantine protocol explain 46% of the variance (*R* = 0.68, *R*^2^ = 0.46, corrected *R*^2^ = 0.46, LL CI_94%_ = 0.40, UL CI_94%_ = 0.51, ω^2^ = 0.46), with social norms being the strongest predictor [Δ*R*^2^ = 0.37, *F*_(1,529)_ = 303.56, *p* < 0.001, LL CI_94%_ = 0.28, UL CI_94%_ = 0.44, ω^2^ = 0.36].

Testing the robustness of the model by bootstrapping, all the predictors identified in stepwise regression analysis were entered in regression analysis using bootstrapping. The results of stepwise regression were supported by using bootstrapping in regression analysis [*F*_(5, 578)_ = 98.95, *p* < 0.001, *R* = 0.68, *R*^2^ = 0.46, corrected *R*^2^ = 0.46, LL CI_94%_ = 0.40, UL CI_94%_ = 0.50, ω^2^ = 46], again with social norms being the strongest predictor [Δ*R*^2^ = 0.37, *F*_(1,582)_ = 333.97, *p* < 0.001, LL CI_94%_ = 0.28, UL CI_94%_ = 0.43, ω^2^ = 0.36] (see Table 4).

**Table 4:** Explaining quarantine adherence at the beginning of quarantine.

		**B**	**SE**	**Beta**	**T**	***p***	**LL CI_**95.5%**_**	**UL CI_**95.5%**_**	***r* (zero order)**
1	(Constant)	0.004	0.021		0.164	0.870	−0.039	0.046	
	zt1_social norms	0.561	0.031	0.604	18.275	0.000	0.499	0.623	0.604
2	(Constant)	0.003	0.021		0.151	0.880	−0.039	0.045	
	zt1_Social norms	0.455	0.035	0.490	13.163	0.000	0.386	0.525	0.604
	zt1_Benefit/effectiveness	0.187	0.031	0.224	6.013	0.000	0.124	0.249	0.473
3	(Constant)	−0.370	0.073		−5.055	0.000	−0.517	−0.223	
	zt1_social norms	0.409	0.035	0.441	11.721	0.000	0.339	0.479	0.604
	zt1_Benefit/effectiveness	0.174	0.030	0.209	5.729	0.000	0.113	0.236	0.473
	zt1_boredom	0.112	0.021	0.176	5.305	0.000	0.069	0.154	0.367
4	(Constant)	−0.360	0.072		−4.978	0.000	−0.505	−0.214	
	zt1_QuSocNorm	0.402	0.034	0.433	11.664	0.000	0.333	0.471	0.604
	zt1_Benefit_Queffective	0.143	0.031	0.172	4.641	0.000	0.081	0.206	0.473
	zt1_Boredom	0.108	0.021	0.171	5.222	0.000	0.067	0.150	0.367
	zt1_Perceived risk Covid	0.126	0.030	0.137	4.210	0.000	0.066	0.187	0.299
5	(Constant)	−0.338	0.072		−4.730	0.000	−0.482	−0.195	
	zt1_QuSocNorm	0.364	0.035	0.392	10.297	0.000	0.293	0.435	0.604
	zt1_Benefit_Queffective	0.132	0.031	0.159	4.322	0.000	0.071	0.194	0.473
	zt1_Boredom	0.102	0.021	0.161	4.961	0.000	0.061	0.143	0.367
	zt1_Perceived risk Covid	0.130	0.030	0.142	4.399	0.000	0.071	0.190	0.299
	zt1_ClearCommunication quarantine protocol	0.145	0.037	0.132	3.974	0.000	0.072	0.219	0.364

*Linear model of predictors with 95.5% BCa CI. CI and SE based on 2,000 bootstrap samples. R^2^ = 0.37 for Step 1, ΔR^2^ = 0.04 for Step 2, ΔR^2^ = 0.03 for Step 3, ΔR^2^ = 0.02 for Step 4, ΔR^2^ = 0.02 for Step 5, (ps <0.001)*.

*Predicting Adherence at the End of Quarantine*. A significant regression equation was found predicting quarantine adherence at the end of quarantine [*F*_(5, 525)_ = 98.50, *p* < 0.001] by the sociodemographic variable age, and by the psychosocial predictors assessed at the beginning of quarantine, social norms, boredom, clear communication of the quarantine protocol and perceived benefit/effectiveness of the quarantine. These variables explained 48% of the variance (*R* = 0.70, *R*^2^ = 0.48, corrected *R*^2^ = 0.48, LL CI_94%_ = 0.42, UL CI_94%_ = 0.53, ω^2^ = 0.48), with social norms being the strongest predictor [Δ*R*^2^ = 0.35 change in *F*_(1,528)_ = 298.21, *p* < 0.001, LL CI_99%_ = 0.28, UL CI_99%_ = 0.43, ω^2^ = 0.36].

Testing the robustness of the model by bootstrapping, the predictors identified were entered into regression analysis. The results were supported when applying bootstrapping in regression analysis [*F*_(5, 578)_ = 108.43, *p* < 0.001], explaining 48% of the variance in quarantine adherence at the end of quarantine (*R* = 0.70, *R*^2^ = 0.48, corrected *R*^2^ = 0.48, LL CI_94%_ = 0.43, UL CI_94%_ = 0.53, ω^2^ = 0.48), with social norms remaining the strongest predictor [Δ*R*^2^ = 0.35, *F*_(1,581)_ = 328.15, *p* < 0.001, LL CI_99%_ = 0.28, UL CI_99%_ = 0.43, ω^2^ = 0.36; see Table 5].

**Table 5:** Predicting quarantine adherence at the end of quarantine.

		**B**	**SE**	**Beta**	**T**	***p***	**LL CI_**95.5%**_**	**UL CI_**95.5%**_**	***r* (zero order)**
1	(Constant)	−0.394	0.119		−3.319	0.001	−0.632	−0.156	
	Age	0.011	0.003	0.140	3.419	0.001	0.005	0.018	0.140
2	(Constant)	−0.342	0.095		−3.600	0.000	−0.533	−0.151	
	Age	0.010	0.003	0.122	3.727	0.000	0.004	0.015	0.140
	zt1_Social norms	0.578	0.032	0.595	18.115	0.000	0.514	0.642	0.599
3	(Constant)	−0.925	0.110		−8.423	0.000	−1.145	−0.704	
	Age	0.008	0.002	0.097	3.139	0.002	0.003	0.013	0.140
	zt1_Social norms	0.486	0.032	0.500	15.377	0.000	0.422	0.549	0.599
	zt1_Boredom	0.196	0.022	0.296	9.060	0.000	0.152	0.239	0.467
4	(Constant)	−0.909	0.108		−8.422	0.000	−1.126	−0.692	
	Age	0.008	0.002	0.101	3.340	0.001	0.003	0.013	0.140
	zt1_Social norms	0.433	0.033	0.446	13.136	0.000	0.367	0.500	0.599
	zt1_Boredom	0.187	0.021	0.283	8.785	0.000	0.144	0.230	0.467
	zt1_clear quarantine protocol	0.176	0.038	0.153	4.665	0.000	0.100	0.251	0.376
5	(Constant)	−0.879	0.107		−8.218	0.000	−1.094	−0.664	
	Age	0.008	0.002	0.097	3.229	0.001	0.003	0.013	0.140
	zt1_Social norms	0.374	0.036	0.385	10.367	0.000	0.302	0.447	0.599
	zt1_Boredom	0.182	0.021	0.275	8.618	0.000	0.140	0.224	0.467
	zt1_clear quarantine protocol	0.164	0.037	0.142	4.376	0.000	0.088	0.239	0.376
	zt1_Effectiveness of quarantine	0.116	0.030	0.133	3.807	0.000	0.055	0.177	0.434

*Linear model of predictors with 95.5% BCa CI. CI and SE based on 2,000 bootstrap samples. R^2^ = 0.02 for Step 1, ΔR^2^ = 0.35 for Step 2, ΔR^2^ = 0.08 for Step 3, ΔR^2^ = 0.02 for Step 4, ΔR^2^ = 0.01 for Step 5, (ps <0.001)*.

*Explaining Adherence at the End of Quarantine*. When entering sociodemographic and psychosocial variable in two steps in stepwise regression, a significant regression equation was found predicting quarantine adherence at the end of quarantine [*F*_(7, 455)_ = 82.266, *p* < 0.001] by the sociodemographic variable age [*R* = 0.14, *R*^2^ = 0.02, corrected *R*^2^ = 0.02, change in *F*_(1,529)_ = 10.63, *p* = 0.001, LL CI_99%_ = 0.0, UL CI_99%_ = 0.06, ω^2^ = 02], and by the psychosocial predictors social norms, boredom, perceived benefit/effectiveness of the quarantine and clear communication of the quarantine protocol. These variables explained 57% of the variance (*R* = 0.76, *R*^2^ = 0.58, corrected *R*^2^ = 0.57, LL CI_94%_ = 0.50, UL CI_94%_ = 0.60, ω^2^ = 0.55), with social norms being the strongest predictor [Δ*R*^2^ = 0.45, change in *F*_(4,459)_ = 334.66, *p* < 0.001, LL CI_94%_ = 0.36, UL CI_94%_ = 0.50, ω^2^ = 0.43].

When sociodemographic and psychosocial variables are entered in one step, the significant regression predicting adherence at the end of quarantine is also predicted by predictors social norms, boredom, perceived benefit/effectiveness of the quarantine, and clear communication of the quarantine protocol [*F*_(5, 525)_ = 145.38, *p* < 0.001, *R* = 0.76, *R*^2^ = 0.58, corrected *R*^2^ = 0.58, LL CI_99%_ = 0.53, UL CI_99%_ = 0.62, ω^2^ = 0.58]. Instead of “age,” “perceived risk of infection” is added to the regression model [Δ*R*^2^ = 0.01, change in *F*_(1,579)_ = 8.85, *p* = 0.003, LL CI_99%_ = 0.00, UL CI_99%_ = 0.05, ω^2^ = 0.01] suggesting that age is a proxy variable for “perceived risk of infection.” Social norms remains the strongest predictor in the equation [Δ*R*^2^ = 0.43, change in *F*_(1,529)_ = 406.27, *p* = 0.000, LL CI_99%_ = 0.36, UL CI_99%_ = 0.50, ω^2^ = 0.43].

Testing the robustness of the model by bootstrapping, the predictors identified were entered in regression analysis. The results were supported by using bootstrapping in regression analysis, [*F*_(5, 579)_ = 151.24, *p* < 0.001], explaining 56% of the variance (*R* = 0.75, *R*^2^ = 0.57, corrected *R*^2^ = 0.56, LL CI_94%_ = 0.52, UL CI_94%_ = 0.60, ω^2^ = 0.56), again with social norms remaining the strongest predictor [Δ*R*^2^ = 0.43, change in *F*_(1,583)_ = 447.73, *p* < 0.001, LL CI_99%_ = 0.36, UL CI_99%_ = 0.50, ω^2^ = 0.43; see Table 6].

**Table 6:** Explaining quarantine adherence at the end of quarantine.

		**B**	**SE**	**Beta**	**T**	***p***	**LL CI_**95.5%**_**	**UL CI_**95.5%**_**	***r* (zero order)**
1	(Constant)	0.000	0.022		−0.020	0.984	−0.045	0.044	
	zt2_social norms	0.621	0.031	0.659	20.156	0.000	0.559	0.683	0.659
2	(Constant)	−0.001	0.021		−0.031	0.975	−0.043	0.041	
	zt2_social norms	0.532	0.031	0.564	17.063	0.000	0.469	0.594	0.659
	zt2_boredom	0.219	0.027	0.266	8.036	0.000	0.164	0.273	0.467
3	(Constant)	−0.001	0.020		−0.025	0.980	−0.040	0.039	
	zt2_social norms	0.436	0.032	0.463	13.551	0.000	0.372	0.501	0.659
	zt2_boredom	0.200	0.026	0.244	7.718	0.000	0.148	0.253	0.467
	zt2_clear quarantine protocol	0.267	0.035	0.248	7.544	0.000	0.196	0.338	0.509
4	(Constant)	0.000	0.019		−0.012	0.990	−0.039	0.038	
	zt2_social norms	0.349	0.034	0.371	10.129	0.000	0.280	0.419	0.659
	zt2_boredom	0.188	0.025	0.229	7.445	0.000	0.137	0.239	0.467
	zt2_clear quarantine protocol	0.243	0.035	0.226	7.044	0.000	0.174	0.312	0.509
	zt2_effectiveness quarantine	0.171	0.029	0.201	5.921	0.000	0.113	0.229	0.534
5	(Constant)	0.000	0.019		−0.012	0.990	−0.039	0.038	
	zt2_social norms	0.346	0.034	0.367	10.086	0.000	0.277	0.414	0.659
	zt2_boredom	0.186	0.025	0.226	7.426	0.000	0.136	0.237	0.467
	zt2_clear quarantine protocol	0.248	0.034	0.231	7.245	0.000	0.180	0.317	0.509
	zt2_effectiveness quarantine	0.148	0.030	0.174	4.970	0.000	0.088	0.208	0.534
	zt2_risk Covid	0.084	0.028	0.089	2.975	0.003	0.027	0.140	0.257

*Linear model of predictors with 95.5% BCa CI. CI and SE based on 2,000 bootstrap samples. R^2^ = 0.43 for Step 1, ΔR^2^ = 0.06 for Step 2, ΔR^2^ = 0.05 for Step 3, ΔR^2^ = 0.03 for Step 4, ΔR^2^ = 0.01 for Step 5, (ps <0.001)*.

Due to stepwise regression the interrelated predictors were either omitted from the regression model or were left to explain a small proportion of the variance. However, relationships of social norms supporting quarantine adherence are associated with quarantine adherence and with the other quarantine-related factors with correlations varying between *r* = 0.33 and *r* = 0.57 (*p* < 0.001, *n* = 566). While the correlations are covered in the Supplementary Material, the most outstanding correlations with social norms supporting quarantine adherence should be cited here: perceived benefit/effectiveness of pre-deployment quarantine (*r* = 0.57), fulfilling the need for bonding/intimacy (*r* = 0.35, *p* < 0.001), clear communication of the quarantine protocol (*r* = 0.45, *p* < 0.001), practicality of the quarantine (*r* = 0.40), no financial disadvantage (*r* = 0.34), boredom (*r* = 0.36) health promoting leadership (*r* = 0.33).

As to changes of quarantine adherence over the period of assessment, February–July 2021, no association was found for quarantine adherence (*r* = −0.03, *p* = 0.213, *n* = 579), but for social norms supporting pre-deployment quarantine (*r* = −0.12, *p* = 0.003, *n* = 579) and for perceived risk of infection (*r* = −0.104, *p* = 0.006, *n* = 579).

## Discussion

This study analyzed (a) if pre-deployment individual quarantine might affect mental health, perceived social support, perceived unit cohesion, (b) if adherence with the quarantine protocol might change during quarantine, and (c) which factors impact on mental health and adherence with the quarantine protocol.

### Mental Health

Mental health at the end of quarantine could only be explained by a percentage up to 20% with the most influential predictor being perceived social support. Mental health at the beginning and at the end of quarantine were explained by general perceived social support and by the fulfilled need for bonding and intimacy during quarantine. Only mental health at the beginning of quarantine was associated with being in a partnership and perceived unit cohesion; while only mental health at the end of quarantine could be partially predicted by age and accumulated days of quarantine and the quarantine-related factors of “clear communication of the quarantine protocol” and “perceived stigma.” Lower perceived resilience in dealing with pandemic-related behavioral restrictions for young adults is in line with large representative analyses by the Covid Snapshot Monitoring (COSMO) (71) and previous reviews (15, 33). The general strong health protective function of perceived social support is in line with several meta-analyses (57, 72) and the only study on military quarantine, post-deployment collective quarantine (12).

In contrast to previous research for civilian quarantining or isolation following a suspected or confirmed infection, we did not find a significant overall deterioration of mental health during pre-deployment quarantine. We also did not find an overall decrease in perceived social support or perceived unit cohesion. However, for two subgroups we found differing trajectories in respect to mental health and the health protective factors of perceived social support and perceived unit cohesion: Mental health slightly deteriorated over the course of pre-deployment quarantine with increasing accumulated days of isolation. Perceived unit cohesion slightly decreased over the course of pre-deployment for middle rank soldiers, while it increased for lower rank soldiers and remained unaffected for higher ranks. Differing from international research highlighting female gender as a risk factor for adverse mental health impacts by the pandemic in general, potentially facilitating cardiovascular diseases (73, 74), and isolation/quarantine in particular (15, 33), we did not find such effects. One potential explanation is that the protective factors of perceived unit cohesion and perceived social support do not differ between the male and female soldiers and that many of the pandemic- and quarantine-related stress factors have been addressed before pre-deployment quarantine or by quarantine management itself.

Previous rapid and systematic reviews (15, 33) found that length of quarantine and isolation itself is associated with mental health. In our study, we did not look at the impact of one single quarantine, as we expected the length of pre-deployment quarantine not to vary extensively and the pandemic had been going on for a year when the data collection started. We analyzed if the accumulated days of quarantine during the pandemic impacted on mental health over the course of pre-deployment quarantine. To our knowledge, this is the first empirical evidence how previous quarantining or isolation experience is influencing the trajectory of mental health and perceived social support over the course of a new quarantine at the same time controlling for the factor of infection-related traumatic experience. As the occasion for pre-deployment quarantining is neither a confirmed infection with Covid nor a contact with an infected person, infection-associated traumatic experience can be excluded as an influencing factor in this case.

Further explanations for the mental health of soldiers overall is not affected by pre-deployment quarantine are (1) that this is a very healthy sample as they are screened for medical fitness pre-deployment, (2) the protective factor of perceived unit cohesion in spite of being individually isolated, and (3) that many of the quarantine-associated conditions associated with mental health effects and quarantine adherence were addressed during pre-deployment quarantine.

The slight deterioration in mental health associated with accumulated days in quarantine are not considered to be alarming at this point. However, this conclusion does not exclude that the mental health of a number of soldiers is seriously affected by pre-deployment quarantine respective an accumulation of stress factors. The longer-term impact on mental health should be followed up in research. Concerning practical implications, preventive measures are recommended including (a) screening for accumulated days of quarantining prior to pre-deployment quarantining and (b) designing compensatory measures facilitating perceived social support for soldiers with previous quarantining experience and facilitating perceived unit cohesion for soldiers with middle ranks. In addition, an observation of longer term-effects on mental health is recommended. While the need for bonding and intimacy can just be partially influenced during individual isolation conditions by providing good coverage for mobile phone connections and long-term holiday planning, the quarantine-related factors boredom and perceived stigma by fellow soldiers could be addressed by health promoting leadership. Though health promoting leadership did not additionally contribute to predicting mental health, it was found to be associated with mental health and positive social norms toward the pre-deployment quarantine and in particular strongly associated with unit cohesion (see Supplementary Materials).

### Quarantine Adherence

Quarantine adherence could be explained up to 58% by positive social norms toward the quarantine, perceived benefit/effectiveness of quarantine, boredom, perceived risk of infection and clear communication on the quarantine protocol. This result is in line with previous international research (16). COSMO kindly supported us with additional calculations with the purpose of contextualizing of our results for the specific military subgroup with the German population for different assessment waves throughout the pandemic (COSMO, University Erfurt on August 17, 2021). The result of age being a proxy for perceived risk of infection with Covid and perceived resilience to quarantining also conforms with results reported by the Covid Snapshot Monitoring (COSMO) in Germany (71), with young adults between 18 and 29 years considering the risk of being severely infected with the Covid-19 virus as substantially lower as well as their own (psychological) resilience (assessment waves 7 through to wave 13, 14/04–27/06/20 based on calculations provided by Universität Erfurt, COSMO, on August 17, 2021).

For the German civilian population, the knowledge that quarantining is an official directive had the strongest relationship with quarantine adherence, being followed by having been infected with Covid-19. The two most prominent predictors of adherence with the quarantine or isolation protocol for the German civilian population do not show variability with military quarantinees, as there is no way to ignore for the military quarantinees that pre-deployment quarantine has been ordered as quarantinees are neither ordered to quarantine because of an infection or having been in close contact with an infected person. Additional manipulation checks do not show any significant correlations between quarantine adherence and having been infected (*r* = −0.048, *p* = 0.077, *n* = 588) nor between perceived infection risk and having been infected (*r* = −0.006, *p* = 0.432, *n* = 536).

Quarantine adherence and predictors for quarantine adherence in Germany depend on the waves of data collection (calculations provided by COSMO, University Erfurt on August 17, 2021). Self-reported 100% self-isolation following symptoms varied between 57 and 32%. One hundred percent adherence with the quarantine regulations (when having had a confirmed contact with a person tested positive for SARS-COV-2) varied between 50 and 43% (data provided by COSMO on 17th August, 21). These changes seem to reflect perceived risk of infection, as adherence varies with incidence and hospitalization rates over the year. This suggests that quarantine adherence and the weight of its predictors also could change over the course of the pandemic for adherence with pre-deployment quarantine.

More surprising was the effect related to gender in light of previous research describing quarantine adherence as higher for female gender by international research (16–18) as well as by the regular Covid-19 Snapshot Monitoring for Germany (71). The initially indeed slightly higher quarantine adherence for female soldiers decreased over the course of quarantine, leveling in with quarantine adherence of male soldiers at the end of quarantine—in spite of female soldiers initially rating practicality and the benefit/effectiveness of pre-deployment quarantine higher than male soldiers. According to our *post-hoc* exploratory analysis, this gender effect most likely can be attributed to correlations with mental health, though no differences for mental health were found for gender.

The most striking result, from our point of view, was that the strongest predictor was “social norms of relevant others supporting pre-deployment quarantine” predicting more than 40% of quarantine adherence.

Based on these results, the following measures are suggested for facilitating quarantine adherence: Relevant partners and family should be involved in pre-deployment quarantine management. Successfully addressing quarantine-related beliefs and behaviors by military leaders is helped by them being perceived as caring for the well-being of their soldiers (health promoting leadership). Special attention should be paid to younger soldiers by military supervisor, older fellow soldiers and eventually military psychologists addressing perceived infection risk, benefit of the quarantine, perceived social support, and the social norms of fellow soldiers.

The questions remains as how to achieve the goal of relevant others supporting the pre-deployment quarantine (protocol). This is easier said than done. Here, the relationships between the predictors might shed some light, in particular the positive relationship of social norms supporting pre-deployment quarantine with (in descending order) perceived benefit/effectiveness of pre-deployment quarantine and the quarantine protocol, bonding needs, clear communication of the quarantine protocol, practicality of the quarantine, no financial disadvantage, less boredom and health promoting leadership, with correlations varying between *r* = 0.3 and *r* = 0.56 (*p* < 0.001, *n* = 566). This suggests that a multifaceted approach addressing these factors, also by the help of health promoting leadership could promote supportive social norms and quarantine adherence. As suggested by health supporting leadership and the fulfillment of bonding need and social norms, this is not only a rational, but an emotional and social endeavor.

However, there might be a limit as to which support for pre-deployment quarantine can be facilitated: Though quarantine adherence did not decrease between February and July 2021 (see Supplementary Material), two of the relevant predictors did: social norms supporting the quarantine and perceived risk of infection. As to the most influential predictors of quarantine adherence, we cannot determine if decreasing perceived risk of infection and perceived positive social norms toward the quarantine over the course of the study period were due to the increasing immunization, decreasing incidence rates, habituation, complacency or a mix of these factors. While we cannot single out the one reason, this development suggests that quarantine adherence will decline in the mid- or longterm as well, in particular due to relevant others, including family/partner and fellow soldiers, becoming more critical toward pre-deployment quarantine. In this light of perceived decreasing support, it is recommended (a) to keep ordered quarantining commensurate. This development could be observed as mandatoriness and length of pre-deployment quarantine have been changed dependent on immunization status, country of deployment, and policies of international organizations (United Nations, NATO). At the same time, deploying soldiers still ordered to quarantine, their families/partners and fellow soldiers might need even clearer leadership communication as to why they have to quarantine and others have not to.

### Limitations and Strengths

This prospective design included a large sample which was close to representative for the troops deploying between February and July 2021. To our knowledge, it is the first prospective study on the impact of quarantining. This particular kind of planned pre-deployment quarantine provided a rare opportunity to control a number of quarantine-related factors resulting in an almost quasi-experimental study: the absence of infection-related traumatic experience, the practicalities, including provision of daily needs and medical care, a 24/7-h hotline, financial disadvantage and compensation for the period of confinement.

Limitations of our study are that we did not ask about actual violations of the quarantine protocol, as receiving knowledge about such transgressions would have obliged us as researchers and military personnel to report breaching the confidentiality of the information and leading to investigations and disruptions of the deployment. The pandemic did not allow for recruiting a control group of soldiers deploying without being quarantined. Missing information on sociodemographic variables partially resulted into excluding up to 130 cases from a sample of 600. Potentially biased results due to these exclusions cannot be fully ruled out. Results for small groups, including female gender and single caretakers have to be regarded with some caution, e.g., the non-significant relation for female soldiers with more adverse mental health than male soldiers at the end of quarantine (*r* = 0.073, *p* = 0.041, *n* = 573; see Supplementary Material), though these groups were not underrepresented when comparing with the percentage of these groups deploying.

During the recruitment period, the inoculation program started resulting in a growing number of partially and fully vaccinated soldiers reaching almost 100% of fully vaccinated deploying soldiers in July 2021. At the point of the study proposal, we expected the pre-deployment quarantine to be discontinued for vaccinated soldiers. Therefore, we did not include questions about vaccinations. Controlling for time of assessment could capture the effect of inoculation as well as a habituation effect in respect to perceived risk of infection or a realistic assessment of decreasing incidence rates with the summer approaching. The social norms toward the quarantine were perceived as less supportive over time; again this could be attributed to vaccinations as well as decreasing incidence rates. For the very slight tendency of quarantine adherence decreasing toward the summer, no significant effect was found.

### Future Research

Summing up avenues for future research, we recommend to follow up on the long-term impact of pre-deployment quarantining on mental health and the protective factors of perceived social support and unit cohesion. The quality of research could be strengthened by including control groups though possibly not during the pandemic and by further validating the assessment instruments, in particular by assessing the associations between the adherence questionnaire with actual violations of the quarantine protocol. Further insights into factors shaping quarantine adherence could be won by comparing military and civilian quarantine.

## Author's Note

Military Hospital Berlin (for regular costs for conducting the study) Medical Academy of the German Armed Forces (Sanitätsakademie der Bundeswehr). Coping with risky deployment abroad requires mental and physical readiness. This study provides first insights into how military pre-deployment quarantine affects mental health and quarantine adherence and its mitigating factors. Studying soldiers' pre-deployment quarantine provides the unique opportunity of a quasi-experimental design. External factors identified to influence mental health and quarantine adherence are controlled for by the military setting, which provides the military quarantinees with regular briefings on Covid and the quarantine, necessary supplies, financial safety and compensation. Quarantine-protocol violations are quite likely to be detected, investigated and to result in disciplinary measures. Foremost, in this quarantine setting, the potential traumatic factor of being infected with a health- or life-threatening disease is absent, thereby allowing to isolate the impact of the quarantine from the impact of the traumatic event. Studies on quarantining and isolation found adverse mental health effects for those in quarantine and isolation based on cross-sectional and retrospective longitudinal designs. To our knowledge, this is the first study with a prospective design analyzing mental health and quarantine adherence over the course of the quarantine, as well as changes in the protective factors perceived social support and its military-specific form, perceived unit cohesion.

## Data Availability Statement

The datasets presented in this article are not readily available because they form a part of an ongoing longitudinal study of which ownership belongs to the German Ministry of Defense. Requests to access the datasets should be directed to the authors, gerddieterwillmund@bundeswehr.org and antjeheikebuehler@bundeswehr.org.

## Ethics Statement

The studies involving human participants were reviewed and approved by Charité Ethics Committee, Ethics' Approval of registered research proposal EA1/388/20. The patients/participants provided their written informed consent to participate in this study.

## Author Contributions

All authors listed have made a substantial, direct, and intellectual contribution to the work and approved it for publication.

## Conflict of Interest

The authors declare that the research was conducted in the absence of any commercial or financial relationships that could be construed as a potential conflict of interest.

## Publisher's Note

All claims expressed in this article are solely those of the authors and do not necessarily represent those of their affiliated organizations, or those of the publisher, the editors and the reviewers. Any product that may be evaluated in this article, or claim that may be made by its manufacturer, is not guaranteed or endorsed by the publisher.

## Footnotes

^1^When the deployment period is shortened, compensatory payment, linked to days deployed, is reduced as well.

## Supplementary Material

### Overview of Supplementary Materials

1. Calculation of Required sample sizes (ad 2.4.1)

Correlations between

2. Sociodemographic factors (table 1)

Correlations between sociodemographic factors and

3. Quarantine adherence and mental health (table 2)
4. Perceived social support (table 3)
5. Perceived unit cohesion (table 4)
6. Health promoting leadership (table 5)
7. Quarantine-related psychosocial factors assessed at the beginning of pre-deployment quarantine (table 6)
8. Quarantine-related psychosocial factors assessed at the end of pre-deployment quarantine (table 7)

Correlations between

9. Quarantine adherence, mental health, perceived social support, perceived unit cohesion and health promoting leadership (table 8)
10. Quarantine-related psychosocial factors assessed at the beginning of quarantine (table 9)
11. Quarantine-related psychosocial factors assessed at the end of quarantine (table 10)
12. Quarantine-related psychosocial factors assessed at the beginning and at the end of quarantine (table 11)

Correlations between

13. Quarantine-related psychosocial factors assessed at the beginning of pre-deployment quarantine on the one hand and quarantine adherence and mental health assessed at the beginning and at the end of pre-deployment quarantine (table 12)
14. Quarantine-related psychosocial factors on the one hand and quarantine adherence and mental health assessed on the other hand, all assessed at the end of pre-deployment quarantine (table 13).

## References

[1] Martini M Gazzaniga V Bragazzi NL Barberis I. The Spanish influenza pandemic: a lesson from history 100 years after 1918. J Prev Med Hyg. (2019) 60:E64–7. 10.15167/2421-4248/jpmh2019.60.1.1205 31041413 PMC6477554

[2] World Health Organization. Pandemic Influenza Risk Management: A WHO Guide to Inform and Harmonize National and International Pandemic Preparedness AND Response. Geneva (2018).

[3] Johnson NP Mueller J. Updating the accounts: global mortality of the 1918-1920 “Spanish” influenza pandemic. Bull Hist Med. (2002) 76:105–15. 10.1353/bhm.2002.0022 11875246

[4] Councell CE. War and infectious disease. Public Health Rep. (1941) 56:547–73.19315809

[5] Spinney L. Pale Rider: The Spanish Flu of 1918 and How it Changed the World. New York, NY: Public Affairs (2018).

[6] U.S. Department of Defense Personnel and Readiness. Force Health Protection Guidance (Supplement 16) - Department of Defense Guidance for Deployment and Redeployment of Individuals and Units During the Coronavirus Disease 2019 Pandemic. Washington, DC (2021). p. 8.

[7] U. S. Department of Defense Personnel and Readiness. Force Health Protection Guidance Supplement 9(Supplement 9): Department of Defense Guidance for Deployment and Redeployment of Individuals and Units during the Novel Coronavirus Disease 2019 Pandemic. Washington, DC (2020). p. 6.

[8] Medical Corps of the German Armed Forces. Fachliche Leitlinie zum Gesundheits-/Infektionsschutz im Rahmen der SARS-CoV-2 Pandemie in der Bundeswehr. Koblenz (2020).

[9] Operations Command of the German Armed Forces. Weisung Nr. 1 für die Durchführung einer isolierten Unterbringung vor Verlegung von Personal in die Einsatz- und Missionsgebiete der Bundeswehr und die Vorbereitung einer Quarantäe nach Rückkehr (COVID-19). Schwielowsee (2020).

[10] Nussbaumer-Streit B Mayr V Dobrescu AI Chapman A Persad E Klerings I. Quarantine alone or in combination with other public health measures to control COVID-19: a rapid review. Cochrane Database Syst Rev. (2020) 4:CD013574. 10.1002/14651858.CD013574 32267544 PMC7141753

[11] Wehrbeauftragte des Deutschen Bundestages. Unterrichtung durch die Wehrbeauftragte: Jahresbericht 2020 (62). Bericht). Berlin (2021). p. 150.

[12] Adler AB Kim PY Thomas SJ Sipos LM. Quarantine and the U.S. military response to the Ebola crisis: soldier health and attitudes: short communication. Public Health. (2018) 155:95–8. 10.1016/j.puhe.2017.11.020 29331771

[13] Sipos ML Kim PY Thomas SJ Adler AB. U.S. service member deployment in response to the Ebola crisis: the psychological perspective. Mil Med. (2018) 183:e171–8. 10.1093/milmed/usx042 29514338

[14] Sundin J Herrell RK Hoge CW Fear NT Adler AB Greenberg N. Mental health outcomes in US and UK military personnel returning from Iraq. Br J Psychiatry. (2014) 204:200–7. 10.1192/bjp.bp.113.129569 24434071

[15] Brooks SK Webster RK Smith LE Woodland L Wessely S Greenberg N. The psychological impact of quarantine and how to reduce it: rapid review of the evidence. Lancet. (2020) 395:912–20. 10.1016/S0140-6736(20)30460-8 32112714 PMC7158942

[16] Webster RK Brooks SK Smith LE Woodland L Wessely S Rubin GJ. How to improve adherence with quarantine: rapid review of the evidence. Public Health. (2020) 182:163–9. 10.1016/j.puhe.2020.03.007 32334182 PMC7194967

[17] Bundesministerium des Innern für Bau und Heimat. Polizeiliche Kriminalstatistik 2020: Ausgewählte Zahlen im Überblick. Berlin (2021).

[18] Eastwood K Durrheim D Francis JL d'Espaignet ET Duncan S Islam F. Knowledge about pandemic influenza and compliance with containment measures among Australians. Bull World Health Organ. (2009) 87:588–94. 10.2471/blt.08.060772 19705008 PMC2733278

[19] Wray RJ Harris JK Jupka K Vijaykumar S Mitchell EW Pollard W. Individual and community influences on adherence to directives in the event of a plague attack: survey results. Disaster Med Public Health Prep. (2012) 6:253–62. 10.1001/dmp.2012.43 23077268

[20] Bauerle Bass S Burt Ruzek S Ward L Gordon TF Hanlon A Hausman AJ. If you ask them, will they come? Predictors of quarantine compliance during a hypothetical avian influenza pandemic: results from a statewide survey. Disaster Med Public Health Prep. (2010) 4:135–44. 10.1001/dmphp.d-09-00052r2 20526136

[21] Taylor M Raphael B Barr M Agho K Stevens G Jorm L. Public health measures during an anticipated influenza pandemic: factors influencing willingness to comply. Risk Manag Healthc Policy. (2009) 2:9–20. 10.2147/RMHP.S4810 22312204 PMC3270909

[22] Murphy ST Cody M Frank LB Glik D Ang A. Predictors of emergency preparedness and compliance. Disaster Med Public Health Prep. (2009) 3:33–41. 10.1097/DMP.0b013e3181a9c6c5 19590429

[23] Kim EY Liao Q Yu ES Kim JH Yoon SW Lam WW. Middle East respiratory syndrome in South Korea during 2015: risk-related perceptions and quarantine attitudes. Am J Infect Control. (2016) 44:1414–6. 10.1016/j.ajic.2016.03.014 27130900 PMC7115318

[24] Reynolds DL Garay JR Deamond SL Moran MK Gold W Styra R. Understanding, compliance and psychological impact of the SARS quarantine experience. Epidemiol Infect. (2008) 136:997–1007. 10.1017/S0950268807009156 17662167 PMC2870884

[25] Al Zabadi H Yaseen N Alhroub T Haj-Yahya M. Assessment of quarantine understanding and adherence to lockdown measures during the COVID-19 pandemic in Palestine: community experience and evidence for action. Front Public Health. (2021) 9:570242. 10.3389/fpubh.2021.570242 33738274 PMC7960769

[26] Pollak Y Dayan H Shoham R Berger I. Predictors of non-adherence to public health instructions during the COVID-19 pandemic. Psychiatry Clin Neurosci. (2020) 74:602–4. 10.1111/pcn.13122 32729646

[27] Tooher R Collins JE Street JM Braunack-Mayer A Marshall H. Community knowledge, behaviours and attitudes about the 2009 H1N1 Influenza pandemic: a systematic review. Influenza Other Respir Viruses. (2013) 7:1316–27. 10.1111/irv.12103 23560537 PMC4634241

[28] Wenzel T. Förderfaktoren und Barrieren für die Quarantäne-Compliance: What do we know? Ein systematisches Review aus dem. Berlin: Projekt Q-Co. (2019).

[29] Baum NM Jacobson PD Goold SD. “Listen to the people”: public deliberation about social distancing measures in a pandemic. Am J Bioeth. (2009) 9:4–14. 10.1080/15265160903197531 19882444

[30] Cava MA Fay KE Beanlands HJ McCay EA Wignall R. Risk perception and compliance with quarantine during the SARS outbreak. J Nurs Scholarsh. (2005) 37:343–7. 10.1111/j.1547-5069.2005.00059.x 16396407

[31] DiGiovanni C Conley J Chiu D Zaborski J. Factors influencing compliance with quarantine in Toronto during the 2003 SARS outbreak. Biosecur Bioterror. (2004) 2:265–72. 10.1089/bsp.2004.2.265 15650436

[32] Smith MJ Bensimon CM Perez DF Sahni SS Upshur RE. Restrictive measures in an influenza pandemic: a qualitative study of public perspectives. Can J Public Health. (2012) 103:e348–52. 10.1007/BF03404439 23617986 PMC6973992

[33] Röhr S Müller F Jung F Apfelbacher C Seidler A Riedel-Heller SG. Psychosocial impact of quarantine measures during serious coronavirus outbreaks: a rapid review. Psychiatr Prax. (2020) 47:179–89. 10.1055/a-1159-5562 32340047 PMC7295307

[34] Henssler J Stock F van Bohemen J Walter H Heinz A Brandt L. Mental health effects of infection containment strategies: quarantine and isolation-a systematic review and meta-analysis. Eur Arch Psychiatry Clin Neurosci. (2021) 271:223–34. 10.1007/s00406-020-01196-x 33025099 PMC7538183

[35] Luo M Guo L Yu M Jiang W Wang H. The psychological and mental impact of coronavirus disease 2019 (COVID-19) on medical staff and general public - a systematic review and meta-analysis. Psychiatry Res. (2020) 291:113190. 10.1016/j.psychres.2020.113190 32563745 PMC7276119

[36] Salari N Hosseinian-Far A Jalali R Vaisi-Raygani A Rasoulpoor S Mohammadi M. Prevalence of stress, anxiety, depression among the general population during the COVID-19 pandemic: a systematic review and meta-analysis. Global Health. (2020) 16:57. 10.1186/s12992-020-00589-w 32631403 PMC7338126

[37] Vindegaard N Benros ME. COVID-19 pandemic and mental health consequences: systematic review of the current evidence. Brain Behav Immun. (2020) 89: 531–42. 10.1016/j.bbi.2020.05.048 32485289 PMC7260522

[38] Prati G Mancini AD. The psychological impact of COVID-19 pandemic lockdowns: a review and meta-analysis of longitudinal studies and natural experiments. Psychol Med. (2021) 51:201–11. 10.1017/S0033291721000015 33436130 PMC7844215

[39] Taylor MR Agho KE Stevens GJ Raphael B. Factors influencing psychological distress during a disease epidemic: data from Australia's first outbreak of equine influenza. BMC Public Health. (2008) 8:347. 10.1186/1471-2458-8-347 18831770 PMC2571100

[40] Wu P Fang Y Guan Z Fan B Kong J Yao Z. The psychological impact of the SARS epidemic on hospital employees in China: exposure, risk perception, and altruistic acceptance of risk. Can J Psychiatry. (2009) 54:302–11. 10.1177/070674370905400504 19497162 PMC3780353

[41] Sprang G Silman M. Posttraumatic stress disorder in parents and youth after health-related disasters. Disaster Med Public Health Prep. (2013) 7:105–10. 10.1017/dmp.2013.22 24618142

[42] Jeong H Yim HW Song Y Ki M Min J Cho J. Mental health status of people isolated due to Middle East Respiratory Syndrome. Epidemiol Health. (2016) 38:e2016048. 10.4178/epih.e2016048 28196409 PMC5177805

[43] Findik UY Ozbaş A Cavdar I Erkan T Topcu SY. Effects of the contact isolation application on anxiety and depression levels of the patients. Int J Nurs Pract. (2012) 18:340–6. 10.1111/j.1440-172X.2012.02049.x 22845633

[44] Mihashi M Otsubo Y Yinjuan X Nagatomi K Hoshiko M Ishitake T. Predictive factors of psychological disorder development during recovery following SARS outbreak. Health Psychol. (2009) 28:91–100. 10.1037/a0013674 19210022

[45] Ko C Yen C Yen J Yang M. Psychosocial impact among the public of the severe acute respiratory syndrome epidemic in Taiwan. Psychiatry Clin Neurosci. (2006) 60:397–403. 10.1111/j.1440-1819.2006.01522.x 16884438

[46] Wu P Liu X Fang Y Fan B Fuller CJ Guan Z. Alcohol abuse/dependence symptoms among hospital employees exposed to a SARS outbreak. Alcohol Alcohol. (2008) 43:706–12. 10.1093/alcalc/agn073 18790829 PMC2720767

[47] Bai Y Lin C Lin C Chen J Chue C Chou P. Survey of stress reactions among health care workers involved with the SARS outbreak. Psychiatr Serv. (2004) 55:1055–7. 10.1176/appi.ps.55.9.1055 15345768

[48] Hawryluck L Gold WL Robinson S Pogorski S Galea S Styra R. SARS control and psychological effects of quarantine, Toronto, Canada. Emerg Infect Dis. (2004) 10:1206–12. 10.3201/eid1007.030703 15324539 PMC3323345

[49] Soon MM Madigan E Jones KR Salata RA. An exploration of the psychologic impact of contact isolation on patients in Singapore. Am J Infect Control. (2013) 41:e111–3. 10.1016/j.ajic.2013.01.037 23663862

[50] Xiao H Zhang Y Kong D Li S Yang N. Social capital and sleep quality in individuals who self-isolated for 14 days during the coronavirus disease 2019 (COVID-19) outbreak in January 2020 in China. Med Sci Monit. (2020) 26:e923921. 10.12659/MSM.923921 32194290 PMC7111105

[51] Pietrzak E Pullman S Cotea C Nasveld P. Effects of deployment on mental health in modern military forces: a review of longitudinal studies. J Milit Veterans Health. (2012) 20:24–36. Available online at: https://jmvh.org/wp-content/uploads/2012/08/JMVH-Vol20-August-2.pdf

[52] Trautmann S Goodwin L Höfler M Jacobi F Strehle J Zimmermann P. Prevalence and severity of mental disorders in military personnel: a standardised comparison with civilians. Epidemiol Psychiatr Sci. (2017) 26:199–208. 10.1017/S204579601600024X 27086743 PMC6998688

[53] Thomas S Hummel KV Schäfer J Wittchen H Trautmann S. The role of harassment and group cohesion for depressive and anxiety symptoms. Can J of Behav Sci. (2021) 53. 10.1037/cbs0000283

[54] Pietrzak RH Johnson DC Goldstein MB Malley JC Rivers AJ Morgan CA. Psychosocial buffers of traumatic stress, depressive symptoms, and psychosocial difficulties in veterans of Operations Enduring Freedom and Iraqi Freedom: the role of resilience, unit support, and postdeployment social support. J Affect Disord. (2010) 120:188–92. 10.1016/j.jad.2009.04.015 19443043

[55] McAndrew LM Markowitz S Lu S Borders A Rothman D Quigley KS. Resilience during war: better unit cohesion and reductions in avoidant coping are associated with better mental health function after combat deployment. Psychol Trauma. (2017) 9:52–61. 10.1037/tra0000152 27455138 PMC6549499

[56] Du Preez J Sundin J Wessely S Fear NT. Unit cohesion and mental health in the UK armed forces. Occup Med. (2012) 62:47–53. 10.1093/occmed/kqr151 22003060

[57] Xue C Ge Y Tang B Liu Y Kang P Wang M. A meta-analysis of risk factors for combat-related PTSD among military personnel and veterans. PLoS ONE. (2015) 10:e0120270. 10.1371/journal.pone.0120270 25793582 PMC4368749

[58] Operations Command of the German Armed Forces. Weisung Nr. 2 für die Festlegung von Einsatz- und Missionsgebieten der Bundeswehr für die isolierte Unterbringung vor und Absonderung nach Einsätzen in DEU aufgrund Covid-19 durchzuführen ist, 12. Änderung. Schwielowsee (2021).

[59] Franke GH. Manual Mini-SCL: Mini-Symptom-Checklist. Göttingen: Hogrefe (2017).

[60] Mahler C Hermann K Horne R Ludt S Haefeli WE Szecsenyi J. Assessing reported adherence to pharmacological treatment recommendations. Translation and evaluation of the Medication Adherence Report Scale (MARS) in Germany. J Eval Clin Pract. (2010) 16:574–9. 10.1111/j.1365-2753.2009.01169.x 20210821

[61] Bühler A Wesemann U Willmund G.-D. Isolierte Unterbringung und häusliche Absonderung – ein Werkzeugkasten zur Untersuchung von Quarantäneadhärenz, quarantäne-assoziierten und militär-spezifischen Einflussfaktoren. Wehrmedizinische Monatsschrift. (Unpublished).

[62] Fydrich T Sommer G Brähler E. Fragebogen zur sozialen Unterstützung: F SozU. Göttingen: Hogrefe (2007).

[63] Dunkel D Antretter E Fröhlich-Walser S Haring C. Evaluation der Kurzform des Fragebogens zur Sozialen Unterstützung (F SozU-K22) in klinischen und nichtklinischen Stichproben. Psychother Psychosom Med Psychol. (2005) 55:266–77. 10.1055/s-2004-834746 15875274

[64] Jäger S Franke GH. Der Fragebogen zur sozialen Unterstützung: Psychometrische Prüfung an einer Stichprobe Studierender. Klinische Diagnostik und Evaluation. Göttingen: Vandenhoeck & Ruprecht (2011) 3:427–46.

[65] Sommer G Fydrich T. Soziale Unterstützung. Diagnostik, Konzepte, F-SozuMaterialien, 22. Tübingen: Deutsche Gesellschaft für Verhaltenstherapie (1989).

[66] Faul F Erdfelder E Lang A Buchner A. G^*^Power 3: a flexible statistical power analysis program for the social, behavioral, and biomedical sciences. Behav Res Methods. (2007) 39:175–91. 10.3758/bf03193146 17695343

[67] Eid M Gollwitzer M Schmitt M editors. Statistik und Forschungsmethoden: Lehrbuch - mit Onlinematerial. Weinheim: Beltz (2017).

[68] Smithson M. Applications in ANOVA and regression. In: Smithson M, editor. Confidence Intervals. Thousand Oaks, CA: Sage (2003). p. 42–66.

[69] Carroll RM Nordholm LA. Sampling characteristics of Kelley's ε and Hays' ω. Educ Psychol Measur. (1975) 35:541–54. 10.1177/001316447503500304

[70] Blake KR Gangestad S. On attenuated interactions, measurement error, and statistical power: guidelines for social and personality psychologists. Pers Soc Psychol Bul. (2020) 46:1702–11. 10.1177/0146167220913363 32208875

[71] Betsch C Wieler LH Habersaat K. Monitoring behavioural insights related to COVID-19. Lancet. (2020) 395:1255–6. 10.1016/S0140-6736(20)30729-7 32247323 PMC7163179

[72] Brewin CR Andrews B Valentine JD. Meta-analysis of risk factors for posttraumatic stress disorder in trauma-exposed adults. J Consult Clin Psychol. (2000) 68:748–66. 10.1037//0022-006x.68.5.748 11068961

[73] Vogel B Acevedo M Appelman Y Bairey Merz CN Chieffo A Figtree GA. The Lancet women and cardiovascular disease commission: reducing the global burden by 2030. Lancet. (2021) 397:2385–438. 10.1016/S0140-6736(21)00684-X 34010613

[74] Mattioli AV Sciomer S Maffei S Gallina S. Lifestyle and stress management in women during COVID-19 pandemic: impact on cardiovascular risk burden. Am J Lifestyle Med. (2021) 15:356–9. 10.1177/1559827620981014 34025328 PMC8120604

