# Supplementary material for: Adherence and Psychosocial Well-Being During Pandemic-Associated Pre-deployment Quarantine
Source: Front Public Health. 2021 Dec 22;9:802180. doi: 10.3389/fpubh.2021.802180 (PMC8727777; doi:10.3389/fpubh.2021.802180)
Supplement: Supplementary file 1 [file Table_1.pdf]

Table 1: Relationship between sociodemographic variables

			Age	Gender	Partner-ship	Number of children	Single care-taker	Children in emergency care	Rank	Days of deployment	Accumulated days in isolation before pre-deployment quarantine
Kendall-Tau-b	Age	r	.198**	.450**	-.130**	-.085*	-.130**	-.085*	.319**	.364**	.011
		p	.000	.000	.000	.014	.000	.014	.000	.000	.719
		n	591	591	576	571	576	571	578	575	552
	Gender	r	-.059	-.159**	-.100*	.052	-.100*	.052	.041	-.083*	-.006
		p	.148	.000	.017	.216	.017	.216	.306	.018	.866
		n	592	592	577	572	577	572	579	576	553
	Partnership	r	1.000	.334**	.072	-.169**	.072	-.169**	.187**	.100**	.056
		p	.	.000	.086	.000	.086	.000	.000	.005	.134
		n	592	588	573	568	573	568	576	572	549
	Number of children	r	.334**	1.000	-.120**	-.341**	-.120**	-.341**	.084*	.255**	.043
		p	.000	.	.002	.000	.002	.000	.024	.000	.213
		n	588	592	577	572	577	572	576	572	550
	Single caretaker	r	.072	-.120**	1.000	.021	1.000	.021	-.044	-.071*	-.015
		p	.086	.002	.	.625	.	.625	.278	.047	.695
		n	573	577	577	567	577	567	562	558	536
	Children in emergency care	r	-.169**	-.341**	.021	1.000	.021	1.000	-.043	-.041	.008
		p	.000	.000	.625	.	.625	.	.289	.258	.840
		n	568	572	567	572	567	572	556	553	531

		Age	Gender	Partner-ship	Number of children	Single care-taker	Children in emergency care	Rank	Days of deployment	Accumulated days in isolation before pre-deployment quarantine
Rank	r	.187**	.084*	-.044	-.043	-.044	-.043	1.000	.065	.026
	p	.000	.024	.278	.289	.278	.289	.	.058	.475
	n	576	576	562	556	562	556	579	559	537
Days of deployment	r	.100**	.255**	-.071*	-.041	-.071*	-.041	.065	1.000	.149**
	p	.005	.000	.047	.258	.047	.258	.058	.	.000
	n	572	572	558	553	558	553	559	576	534
Accumulated days in quarantine before pre-deployment quarantine	r	.056	.043	-.015	.008	-.015	.008	.026	.149**	1.000
	p	.719	.866	.695	.840	.695	.840	.475	.000	.
	n	552	553	536	531	536	531	537	534	560

*p < .05, ** p < .001

Legend: Coding of sociodemographic variables:

Gender: 1= male, 2= female

Partnership: 1= no, 2= yes

Children in emergency care (parents in occupations with systemic importance during the pandemic can/have to leave their children in pandemic-specific emergency care): 1= yes, 2= no

Single caretaker: 1= yes, 2= no
