# Supplementary material for: Adherence and Psychosocial Well-Being During Pandemic-Associated Pre-deployment Quarantine
Source: Front Public Health. 2021 Dec 22;9:802180. doi: 10.3389/fpubh.2021.802180 (PMC8727777; doi:10.3389/fpubh.2021.802180)
Supplement: Supplementary file 2 [file Table_2.pdf]

**Table 2:** Relationship between mental health (Mini-SCL) and sociodemographic variables (All values of the Mini-SCL and adherence were z-standardized.)

| | | | Mini-SCL t1[1] | Mini-SCL t2[2] | Adherence t1[1] | Adherence t2[2] |
|---|---|---|---|---|---|---|
| Kendall-Tau-b | Mini-SCL t1 | r | 1.000 | .510*** | 1.000 | .592 |
| | | p | . | .000 | . | .000 |
| | | n | 591 | 579 | 597 | 592 |
| | Mini-SCL t2 | r | .510*** | 1.000 | .592 | 1.000 |
| | | p | .000 | . | .000 | . |
| | | n | 579 | 591 | 592 | 598 |
| | Age | r | -.081** | -.091** | .090** | .100*** |
| | | p | .004 | .001 | .001 | .000 |
| | | n | 586 | 583 | 592 | 591 |
| | Gender | r | .043 | .074* | .058 | -.002 |
| | | p | .211 | .032 | .087 | .945 |
| | | n | 587 | 584 | 593 | 592 |
| | Partnership | r | -.101 | -.032 | .001 | .007 |
| | | p | .003 | .359 | .967 | .830 |
| | | n | 583 | 580 | 589 | 588 |
| | Number of children | r | -.081* | -.053 | .048 | .043 |
| | | p | .011 | .100 | .123 | .178 |
| | | n | 583 | 580 | 589 | 588 |
| | Single caretaker | r | -.064 | -.012 | .051 | .060 |
| | | p | .068 | .734 | .134 | .083 |
| | | n | 568 | 565 | 574 | 573 |
| | Children in emergency care | r | .028 | .003 | .042 | .028 |
| | | p | .425 | .929 | .225 | .411 |
| | | n | 563 | 560 | 569 | 568 |
| | Rank | r | -.017 | -.009 | .067 | .079 |
| | | p | .619 | .798 | .041 | .016 |
| | | n | 572 | 567 | 576 | 575 |
| | Days of deployment | r | -.085** | -.087** | .009 | .028 |
| | | p | .004 | .003 | .752 | .342 |
| | | n | 567 | 565 | 573 | 572 |
| | Accumulated days in isolation before pre-deployment quarantine | r | -.020 | -.007 | .063 | -.008 |
| | | p | .511 | .828 | .040 | .789 |
| | | n | 548 | 548 | 554 | 555 |

*p < .05, **p < .01, ***p < .001

[1]t1= beginning of pre-deployment quarantine, [2]t2 = end of pre-deployment quarantine

Legend: Coding of sociodemographic variables:

Gender: 1= male, 2= female

Partnership: 1= no, 2= yes

Single caretaker: 1= yes, 2= no

Children in emergency care (parents in occupations with systemic importance during the pandemic can/have to leave their children in pandemic-specific emergency care): 1= yes, 2= no
