# Supplementary material for: Adherence and Psychosocial Well-Being During Pandemic-Associated Pre-deployment Quarantine
Source: Front Public Health. 2021 Dec 22;9:802180. doi: 10.3389/fpubh.2021.802180 (PMC8727777; doi:10.3389/fpubh.2021.802180)
Supplement: Supplementary file 3 [file Table_3.pdf]

Table 3: Relationship (Kendall-Tau-b) between perceived social support (F-SoZu) and sociodemographic variables (Means of FSoZu based on z-standardized items.)

		F-SoZu t1[1]	F-SoZu t2[2]
F-SoZu t1	r	1.000	.594***
	p	.	.000
	n	593	578
F-SoZu t2	r	.594***	1.000
	p	.000	.
	n	578	588
Age	r	-.077**	-.045
	p	.006	.110
	n	588	580
Gender	r	.075*	.059
	p	.027	.082
	n	589	581
Partnership	r	.045	.056
	p	.188	.101
	n	585	577
Number of children	r	-.025	.015
	p	.433	.642
	n	585	577
Single caretaker	r	-.016	.025
	p	.645	.473
	n	570	562
Children in emergency care	r	-.036	-.050
	p	.296	.148
	n	565	557
Rank	r	-.049	-.048
	p	.140	.149
	n	573	564
Days of deployment	r	-.034	-.027
	p	.239	.353
	n	569	563
Accumulated days in isolation before pre-deployment quarantine	r	-.014	-.048
	p	.649	.123
	n	550	546

*p < .05, **p < .01, ***p < .001

[1]t1= beginning of pre-deployment quarantine, [2]t2 = end of pre-deployment quarantine

Legend: Coding of sociodemographic variables:

Gender: 1= male, 2= female

Partnership: 1= no, 2= yes

Single caretaker: 1= yes, 2= no

Children in emergency care (parents in occupations with systemic importance during the pandemic can/have to leave their children in pandemic-specific emergency care): 1= yes, 2= no
