# Supplementary material for: Adherence and Psychosocial Well-Being During Pandemic-Associated Pre-deployment Quarantine
Source: Front Public Health. 2021 Dec 22;9:802180. doi: 10.3389/fpubh.2021.802180 (PMC8727777; doi:10.3389/fpubh.2021.802180)
Supplement: Supplementary file 4 [file Table_4.pdf]

Table 4: Relationship between perceived unit cohesion (unit cohesion) and sociodemographic variables (The means of „perceived unit cohesion" are based on z-standardized item values.)

			Unit Cohesion t1[1]	Unit Cohesion t2[2]
Kendall-Tau-b	Unit Cohesion t1	r	1.000	.578***
		p	.	.000
		n	576	562
	Unit Cohesion t2	r	.578***	1.000
		p	.000	.
		n	562	589
	Age	r	-.007	.040
		p	.817	.163
		n	571	581
	Gender	r	.003	.005
		p	.942	.873
		n	572	582
	Partnership	r	-.071*	-.028
		p	.041	.414
		n	569	578
	Number of children	r	-.066*	.009
		p	.039	.774
		n	568	578
	Single caretaker	r	.024	.041
		p	.489	.239
		n	554	563
	Children in emergency care	r	.005	-.068
		p	.887	.051
		n	548	558
	Rank	r	.031	-.005
		p	.350	.889
		n	556	565
	Accumulated days of deployment	r	-.022	.007
		p	.452	.821
		n	554	564
	Accumulated days in isolation before pre-deployment quarantine	r	-.056	-.073*
		p	.071	.019
		n	533	546

*p < .05, **p < .01, ***p < .001

[1]t1= beginning of pre-deployment quarantine, [2]t2 = end of pre-deployment quarantine

Legend: Coding of sociodemographic variables:
Gender: 1= male, 2= female
Partnership: 1= no, 2= yes
Single caretaker: 1= yes, 2= no
Children in emergency care (parents in occupations with systemic importance during the pandemic can/have to leave their children in pandemic-specific emergency care): 1= yes, 2= no
