# Supplementary material for: Adherence and Psychosocial Well-Being During Pandemic-Associated Pre-deployment Quarantine
Source: Front Public Health. 2021 Dec 22;9:802180. doi: 10.3389/fpubh.2021.802180 (PMC8727777; doi:10.3389/fpubh.2021.802180)
Supplement: Supplementary file 5 [file Table_5.pdf]

Table 5: Relationship between health promoting leadership and sociodemographic variables (All item values of health promoting leadership were z-standardized.)

			Health promoting leadership t1[1]	Health promoting leadership t2[2]
Kendall-Tau-b	Health promoting leadership t1	r	1.000	.627***
		p	.	.000
		n	593	580
	Health promoting leadership t2	r	.627***	1.000
		p	.000	.
		n	580	589
	Age	r	-.056	-.025
		p	.052	.383
		n	588	581
	Gender	r	.013	.015
		p	.705	.660
		n	589	582
	Partnership	r	-.060	-.057
		p	.081	.103
		n	585	578
	Number of children	r	-.089**	-.040
		p	.006	.219
		n	585	578
	Single caretaker	r	.090*	.067
		p	.010	.057
		n	570	565
	Children in emergency care	r	-.011	-.052
		p	.765	.144
		n	565	560
	Rank	r	-.073*	-.053
		p	.030	.118
		n	572	565
	Days of deployment	r	-.120***	-.083**
		p	.000	.005
		n	570	563
	Accumulated days in isolation before pre-deployment quarantine	r	-.053	-.077*
		p	.089	.014
		n	550	546

*p < .05, **p < .01, ***p < .001

[1]t1= beginning of pre-deployment quarantine, [2]t2 = end of pre-deployment quarantine

Legend: Coding of sociodemographic variables:
Gender: 1= male, 2= female
Partnership: 1= no, 2= yes
Single caretaker: 1= yes, 2= no
Children in emergency care (parents in occupations with systemic importance during the pandemic can/have to leave their children in pandemic-specific emergency care): 1= yes, 2= no
