# Supplementary material for: Adherence and Psychosocial Well-Being During Pandemic-Associated Pre-deployment Quarantine
Source: Front Public Health. 2021 Dec 22;9:802180. doi: 10.3389/fpubh.2021.802180 (PMC8727777; doi:10.3389/fpubh.2021.802180)
Supplement: Supplementary file 6 [file Table_6.pdf]

Table 6: Relationship between sociodemographic variables and factors assumed to influence quarantine adherence and mental health assessed at the beginning of pre-deployment quarantine
(All item values of the quarantine-related factors were z-standardized.)

Beginning of pre-deployment quarantine		[1]InfoCovid	[2]Clear Protocol	[3]Social norms	[4]Stigma	[5]Covid risk	[6]Practicality	[7] Bonding need	[8]Boredom	[9]Effective-ness Quarantine	[10]Financial disadvantage
Age	r	.032	.001	.016	-.127***	.140***	.022	.061*	.059*	.029	.116*
	p	.255	.977	.575	.000	.000	.434	.032	.042	.313	.001
	n	588	588	587	582	589	582	587	587	588	588
Gender	r	.048	.119***	.044	.051	.013	.120***	.023	.028	.103**	-.082*
	p	.155	.000	.192	.158	.694	.000	.491	.426	.003	.042
	n	589	589	588	583	590	583	588	588	589	589
Partnership	r	.006	.011	-.122***	-.039	.010	-.040	-.078*	-.049	-.022	.057
	p	.858	.743	.000	.286	.769	.252	.022	.157	.530	.155
	n	585	585	584	579	586	579	584	584	585	585
Number of children	r	.033	-.039	-.067	-.094**	.032	-.049	-.028	-.058	.003	.208***
	p	.294	.215	.033	.005	.316	.126	.375	.075	.927	.000
	n	585	585	584	579	586	579	584	584	585	585
Single caretaker	r	-.001	.067	.071*	.023	.000	.047	.046	.007	.077*	.050
	p	.967	.053	.040	.526	.997	.179	.180	.839	.027	.221
	n	570	570	569	564	571	564	569	570	570	570
Children in emergency care	r	.050	.051	.110**	.062	-.001	.073*	.090*	.091*	.019	-.162***
	p	.150	.141	.001	.093	.969	.039	.009	.010	.592	.000
	n	565	565	564	559	566	559	564	565	565	565

Rank	r	.017	-.007	.021	-.199***	.193***	.043	.025	.124***	.007	-.019
	p	.616	.823	.526	.000	.000	.206	.456	.000	.826	.618
	n	573	573	572	568	574	566	571	571	573	572
Days of deployment	r	.002	-.010	-.051	-.041	.072*	-.042	.042	-.008	-.061*	.161***
	p	.952	.734	.078	.195	.014	.159	.147	.796	.041	.000
	n	570	569	568	564	570	564	568	568	569	569
Accumulated days in isolation before pre-deployment quarantine	r	-.024	-.026	-.004	-.050	.055	.006	-.014	-.026	-.005	.071
	p	.440	.393	.905	.131	.073	.855	.649	.402	.863	.051
	n	550	550	549	544	551	546	550	552	550	550

*p < .05, **p < .01, ***p < .001

Legend:

Horizontal

[1]InfoCovid: feeling well informed about Covid-19

[2]Clear Protocol: clear communication about the quarantine protocol (purpose, lenths, rules, etc.)

[3]Social norms: Positive social norms of relevant others towards the quarantine (family, partner, fellow soldiers)

[4]Stigma: perceived stigma due tot he quarantine

[5]Covid risk: perceived risk by Covid-19 (self, family/partner, fellow sodliers, general)

[6]Practicality: being provided with everything needed during quarantine (daily nccessities, food, medical support)

[8]Boredom: quarantine-related boredom

[9]Effectiveness Quarantine: perceived benefit/effectiveness of quarantine (to protect self, family, fellow soldiers, vulnerable people, preventin deaths)

[10]Financial disadvantage: financial disadvantages caused by quarantining (additional costs for child-care, etc.)

Vertical

Coding of sociodemographic variables:

Gender: 1= male, 2= female

Partnership: 1= no, 2= yes

Single caretaker: 1= yes, 2= no

Children in emergency care (parents in occupations with systemic importance during the pandemic can/have to leave their children in pandemic-specific emergency care): 1= yes, 2= no
