# Supplementary material for: Adherence and Psychosocial Well-Being During Pandemic-Associated Pre-deployment Quarantine
Source: Front Public Health. 2021 Dec 22;9:802180. doi: 10.3389/fpubh.2021.802180 (PMC8727777; doi:10.3389/fpubh.2021.802180)
Supplement: Supplementary file 7 [file Table_7.pdf]

Table 7: Relationship between sociodemographic variables and quarantine-related psychosocial factors assessed at the end of pre-deployment quarantine (All item values of the quarantine-related factors were z-standardized.)

Beginning of pre-deployment quarantine		[1]InfoCovid	[2]Clear Protocol	[3]Social norms	[4]Stigma	[5]Covid risk	[6]Practicality	[7] Bonding need	[8]Boredom	[9]Effective-ness Quarantine	[10]Financial disadvantage
Age	r	.062*	.060*	.023	-.068*	.130***	.022	.058*	.050	.083**	.096
	p	.029	.034	.417	.023	.000	.434	.041	.075	.004	.004
	n	593	593	590	591	591	582	590	587	593	586
Gender	r	.038	.054	.051	.039	-.011	.120***	.049	.026	.042	-.141***
	p	.260	.114	.128	.281	.752	.000	.150	.440	.224	.000
	n	594	594	591	592	592	583	591	588	594	587
Partnership	r	-.010	-.012	-.091	-.043	.013	-.040	-.088**	-.055	-.024	.063
	p	.780	.725	.007	.230	.693	.252	.009	.108	.496	.118
	n	590	590	587	588	588	579	587	584	590	583
Number of children	r	.026	.022	-.062*	-.070*	.032	-.049	-.047	-.059	.032	.164***
	p	.415	.478	.048	.036	.306	.126	.136	.062	.322	.000
	n	590	590	587	588	588	579	587	584	590	583
Single caretaker	r	.012	.038	.077*	.020	-.007	.047	.062	.009	.062	-.088*
	p	.736	.272	.025	.576	.832	.179	.072	.789	.075	.030
	n	575	575	572	573	573	564	572	570	575	569
Children in emergency care	r	.021	.050	.092**	.021	.035	.073*	.075*	.091**	.041	-.106*
	p	.554	.147	.008	.563	.310	.039	.029	.008	.247	.010
	n	570	570	567	568	568	559	567	565	570	564
Rank	r	.037	-.016	.018	-.166***	.196***	.043	.018	.118***	.034	-.049
	p	.264	.624	.584	.000	.000	.206	.586	.000	.313	.207

	n	577	577	574	575	575	566	574	571	577	570
Days of deployment	r	.014	.043	-.047	-.016	.053	-.042	.050	-.011	-.004	.097**
	p	.621	.141	.103	.610	.071	.159	.086	.706	.904	.005
	n	574	574	571	572	573	564	571	568	574	567
Accumulated days in isolation before pre-deployment quarantine	r	.062*	.060*	.023	-.068*	.130***	.022	.058*	.050	.083**	.096**
	p	.029	.034	.417	.023	.000	.434	.041	.075	.004	.004
	n	593	593	590	591	591	582	590	587	593	586

*p < .05, **p < .01, ***p < .001

Legend:

Horizontal: Quarantine-related psychosocial variables

[1]InfoCovid: feeling well informed about Covid-19

[2]Clear Protocol: clear communication about the quarantine protocol (purpose, lenths, rules, etc.)

[3]Social norms: Positive social norms of relevant others towards the quarantine (family, partner, fellow soldiers)

[4]Stigma: perceived stigma due tot he quarantine

[5]Covid risk: perceived risk by Covid-19 (self, family/partner, fellow sodliers, general)

[6]Practicality: being provided with everything needed during quarantine (daily nccessities, food, medical support)

[8]Boredom: quarantine-related boredom

[9]Effectiveness Quarantine: perceived benefit/effectiveness of quarantine (to protect self, family, fellow soldiers, vulnerable people, preventin deaths)

[10]Financial disadvantage: financial disadvantages caused by quarantining (additional costs for child-care, etc.)

Vertical: Coding of sociodemographic variables:

Gender: 1= male, 2= female

Partnership: 1= no, 2= yes

Children in emergency care: 1= yes, 2= no

Single caretaker: 1= yes, 2= no
