# Supplementary material for: Adherence and Psychosocial Well-Being During Pandemic-Associated Pre-deployment Quarantine
Source: Front Public Health. 2021 Dec 22;9:802180. doi: 10.3389/fpubh.2021.802180 (PMC8727777; doi:10.3389/fpubh.2021.802180)
Supplement: Supplementary file 8 [file Table_8.pdf]

Table 8: Relationship between quarantine adherence, mental health, perceived social support, perceived unit cohesion and perceived health-promoting leadership, at the beginning and at the end of pre-deployment quarantine
(All item values of the quarantine-related factors were z-standardized.)

		Adherence at the beginning of quarantine	Adherence at the end of quarantine	zt1_Mini_SCL	zt2_Mini_SCL	zt1_SocialSupport	zt2_SocialSupport	zt1_UnitCohesion	zt2_UnitCohesion	zt1_HealthPromotLeader	zt2_HealthPromotLeader
Adherence at the beginning of quarantine	r	1	,774	-,170	-,186	-,018	,011	,122	,180	,216	,214
	p		,000	,000	,000	,330	,400	,002	,000	,000	,000
	n	597	592	590	585	593	582	576	583	593	583
Adherence at the end of quarantine	r	,774	1	-,155	-,303	,050	,078	,148	,177	,257	,275
	p	,000		,000	,000	,112	,030	,000	,000	,000	,000
	n	592	598	586	588	589	585	571	586	588	586
zt1_Mini_SCL	r	-,170	-,155	1	,417	-,189	-,151	-,234	-,213	-,108	-,153
	p	,000	,000		,000	,000	,000	,000	,000	,005	,000
	n	590	586	591	579	589	576	572	577	587	577
zt2_Mini_SCL	r	-,186	-,303	,417	1	-,192	-,253	-,164	-,214	-,095	-,212
	p	,000	,000	,000		,000	,000	,000	,000	,011	,000
	n	585	588	579	591	581	587	564	586	582	585
zt1_SocialSupport	r	-,018	,050	-,189	-,192	1	,828	,237	,255	,163	,169
	p	,330	,112	,000	,000		,000	,000	,000	,000	,000
	n	593	589	589	581	593	578	575	579	590	579
zt2_SocialSupport	r	,011	,078	-,151	-,253	,828	1	,193	,270	,107	,173
	p	,400	,030	,000	,000	,000		,000	,000	,005	,000
	n	582	585	576	587	578	588	561	585	579	582
zt1_UnitCohesion	r	,122	,148	-,234	-,164	,237	,193	1	,678	,454	,468
	p	,002	,000	,000	,000	,000	,000		,000	,000	,000
	n	576	571	572	564	575	561	576	562	574	562

zt2_UnitCohesion	r	,180	,177	-,213	-,214	,255	,270	,678	1	,440	,568
	p	,000	,000	,000	,000	,000	,000	,000		,000	,000
	n	583	586	577	586	579	585	562	589	579	582
zt1_HealthPromotLeader	r	,216	,257	-,108	-,095	,163	,107	,454	,440	1	,779
	p	,000	,000	,005	,011	,000	,005	,000	,000		,000
	n	593	588	587	582	590	579	574	579	593	580
zt2_HealthPromotLeader	r	,214	,275	-,153	-,212	,169	,173	,468	,568	,779	1
	p	,000	,000	,000	,000	,000	,000	,000	,000	,000	
	n	583	586	577	585	579	582	562	582	580	589

*p < .05, **p < .01, ***p < .001

[1]zt1= beginning of pre-deployment quarantine, [2]zt2 = end of pre-deployment quarantine
