# Supplementary material for: Adherence and Psychosocial Well-Being During Pandemic-Associated Pre-deployment Quarantine
Source: Front Public Health. 2021 Dec 22;9:802180. doi: 10.3389/fpubh.2021.802180 (PMC8727777; doi:10.3389/fpubh.2021.802180)
Supplement: Supplementary file 9 [file Table_9.pdf]

Table 9: Relationship between quarantine-related psychosocialfactors assessed at the beginning of pre-deployment quarantine (All item values of the quarantine-related factors were z-standardized.)

Korrelationen

		[1]Info Covid	[2]Clear Protocol	[3]Social norms	[4]Stigma	[5]Covid risk	[6]Practicality	[7] Bonding need	[8]Boredom	[9]Effective-ness Quarantine	[10]Financial disadvantage
[1]Info Covid	r	1	.281**	.276**	.169**	.133**	.320**	.160**	.187**	.363**	.045
	p		.000	.000	.000	.001	.000	.000	.000	.000	.138
	n	593	592	591	585	592	583	588	585	591	589
[2]Clear Protocol	r	.281**	1	.378**	.248**	.070*	.485**	.205**	.196**	.264**	.195**
	p	.000		.000	.000	.045	.000	.000	.000	.000	.000
	n	592	593	591	585	592	583	588	585	591	589
[3]Social norms	r	.276**	.378**	1	.188**	.206**	.414**	.362**	.324**	.508**	.216**
	p	.000	.000		.000	.000	.000	.000	.000	.000	.000
	n	591	591	592	585	591	582	587	584	590	588
[4]Stigma	r	.169**	.248**	.188**	1	-.028	.214**	.160**	.091*	.168**	.286**
	p	.000	.000	.000		.246	.000	.000	.014	.000	.000
	n	585	585	585	587	586	581	583	579	585	583
[5]Covid risk	r	.133**	.070*	.206**	-.028	1	.068	.003	.117**	.308**	-.047
	p	.001	.045	.000	.246		.051	.475	.002	.000	.125
	n	592	592	591	586	594	585	590	586	593	591
[6]Practicality	r	.320**	.485**	.414**	.214**	.068	1	.321**	.399**	.352**	.220**
	p	.000	.000	.000	.000	.051		.000	.000	.000	.000
	n	583	583	582	581	585	587	587	579	584	586
[7] Bonding need	r	.160**	.205**	.362**	.160**	.003	.321**	1	.303**	.211**	.173**
	p	.000	.000	.000	.000	.475	.000		.000	.000	.000

	n	588	588	587	583	590	587	592	584	589	590
[8]Boredom	r	.187**	.196**	.324**	.091*	.117**	.399**	.303**	1	.227**	.149**
	p	.000	.000	.000	.014	.002	.000	.000		.000	.000
	n	585	585	584	579	586	579	584	595	585	585
[9]Effective-	r	.363**	.264**	.508**	.168**	.308**	.352**	.211**	.227**	1	.098**
ness Quarantine	p	.000	.000	.000	.000	.000	.000	.000	.000		.009
	n	591	591	590	585	593	584	589	585	593	590
[10]Financial	r	.045	.195**	.216**	.286**	-.047	.220**	.173**	.149**	.098**	1
disadvantage	p	.138	.000	.000	.000	.125	.000	.000	.000	.009	
	n	589	589	588	583	591	586	590	585	590	593

*p < .05, **p < .01, ***p < .001

Legend:

Quarantine-related psychosocial variables

[1]InfoCovid: feeling well informed about Covid-19

[2]Clear Protocol: clear communication about the quarantine protocol (purpose, lenths, rules, etc.)

[3]Social norms: Positive social norms of relevant others towards the quarantine (family, partner, fellow soldiers)

[4]Stigma: perceived stigma due tot he quarantine

[5]Covid risk: perceived risk by Covid-19 (self, family/partner, fellow sodliers, general)

[6]Practicality: being provided with everything needed during quarantine (daily nccessities, food, medical support)

[8]Boredom: quarantine-related boredom

[9]Effectiveness Quarantine: perceived benefit/effectiveness of quarantine (to protect self, family, fellow soldiers, vulnerable people, preventin deaths)

[10]Financial disadvantage: financial disadvantages caused by quarantining (additional costs for child-care, etc.)
