# Supplementary material for: Adherence and Psychosocial Well-Being During Pandemic-Associated Pre-deployment Quarantine
Source: Front Public Health. 2021 Dec 22;9:802180. doi: 10.3389/fpubh.2021.802180 (PMC8727777; doi:10.3389/fpubh.2021.802180)
Supplement: Supplementary file 10 [file Table_10.pdf]

Table 10: Relationship between quarantine-related psychsocialfactors assessed at the end of pre-deployment quarantine (All item values of the quarantine-related factors were z-standardized.)

Correlations

		[1]Info Covid	[2]Clear Protocol	[3]Social norms	[4]Stigma	[5]Covid risk	[6]Practicality	[7] Bonding need	[8]Boredom	[9]Effective-ness Quarantine	[10]Financial disadvantage
[1]Info Covid	r	1	.346**	.293**	.150**	.147**	.276**	.203**	.208**	.411**	.090*
	p		.000	.000	.000	.000	.000	.000	.000	.000	.014
	n	601	601	598	599	599	585	598	595	601	594
[2]Clear Protocol	r	.346**	1	.440**	.304**	.070*	.434**	.213**	.234**	.328**	.214**
	p	.000		.000	.000	.043	.000	.000	.000	.000	.000
	n	601	601	598	599	599	585	598	595	601	594
[3]Social norms	r	.293**	.440**	1	.298**	.194**	.396**	.351**	.357**	.533**	.343**
	p	.000	.000		.000	.000	.000	.000	.000	.000	.000
	n	598	598	598	596	597	582	596	592	598	592
[4]Stigma	r	.150**	.304**	.298**	1	-.017	.303**	.243**	.154**	.153**	.333**
	p	.000	.000	.000		.336	.000	.000	.000	.000	.000
	n	599	599	596	599	597	583	596	593	599	592
[5]Covid risk	r	.147**	.070*	.194**	-.017	1	.024	.008	.109**	.321**	-.028
	p	.000	.043	.000	.336		.279	.424	.004	.000	.248
	n	599	599	597	597	599	583	597	593	599	593
[6]Practicality	r	.276**	.434**	.396**	.303**	.024	1	.263**	.399**	.362**	.255**
	p	.000	.000	.000	.000	.279		.000	.000	.000	.000
	n	585	585	582	583	583	587	582	579	585	578
[7] Bonding need	r	.203**	.213**	.351**	.243**	.008	.263**	1	.320**	.218**	.251**
	p	.000	.000	.000	.000	.424	.000		.000	.000	.000

	n	598	598	596	596	597	582	598	594	598	594
[8]Boredom	r	.208**	.234**	.357**	.154**	.109**	.399**	.320**	1	.264**	.176**
	p	.000	.000	.000	.000	.004	.000	.000		.000	.000
	n	595	595	592	593	593	579	594	595	595	594
[9]Effective-ness Quarantine	r	.411**	.328**	.533**	.153**	.321**	.362**	.218**	.264**	1	.132**
	p	.000	.000	.000	.000	.000	.000	.000	.000		.001
	n	601	601	598	599	599	585	598	595	601	594
[10]Financial disadvantage	r	.090*	.214**	.343**	.333**	-.028	.255**	.251**	.176**	.132**	1
	p	.014	.000	.000	.000	.248	.000	.000	.000	.001	
	n	594	594	592	592	593	578	594	594	594	594

*p < .05, **p < .01, ***p < .001

Legend:

Quarantine-related psychosocial variables

[1]InfoCovid: feeling well informed about Covid-19

[2]Clear Protocol: clear communication about the quarantine protocol (purpose, lenths, rules, etc.)

[3]Social norms: Positive social norms of relevant others towards the quarantine (family, partner, fellow soldiers)

[4]Stigma: perceived stigma due tot he quarantine

[5]Covid risk: perceived risk by Covid-19 (self, family/partner, fellow sodliers, general)

[6]Practicality: being provided with everything needed during quarantine (daily nccessities, food, medical support)

[8]Boredom: quarantine-related boredom

[9]Effectiveness Quarantine: perceived benefit/effectiveness of quarantine (to protect self, family, fellow soldiers, vulnerable people, preventin deaths)

[10]Financial disadvantage: financial disadvantages caused by quarantining (additional costs for child-care, etc.)
