# Supplementary material for: Adherence and Psychosocial Well-Being During Pandemic-Associated Pre-deployment Quarantine
Source: Front Public Health. 2021 Dec 22;9:802180. doi: 10.3389/fpubh.2021.802180 (PMC8727777; doi:10.3389/fpubh.2021.802180)
Supplement: Supplementary file 11 [file Table_11.pdf]

Table 11: Relationship of quarantine-related psychosocialfactors between beginning and end of pre-deployment quarantine (All item values of the quarantine-related factors were z-standardized.)

		End of quarantine (t2)									
**Beginning of quarantine (t1)**		[1]Info Covid	[2]Clear Protocol	[3]Social norms	[4]Stigma	[5]Covid risk	[6]Practicality	[7] Bonding need	[8]Boredom	[9]Effective-ness Quarantine	[10]Financial disadvantage
[1]Info Covid	r	.711	.274	.254	.108	.132	.320	.128	.187	.355	.054
	p	.000	.000	.000	.004	.001	.000	.001	.000	.000	.096
	n	591	591	588	589	589	583	588	585	591	584
[2]Clear Protocol	r	.264	.608	.353	.267	.005	.485	.206	.196	.285	.147
	p	.000	.000	.000	.000	.452	.000	.000	.000	.000	.000
	n	591	591	588	589	589	583	588	585	591	584
[3]Social norms	r	.269	.337	.778	.197	.196	.414	.284	.325	.465	.303
	p	.000	.000	.000	.000	.000	.000	.000	.000	.000	.000
	n	590	590	587	589	588	582	587	584	590	583
[4]Stigma	r	.100	.185	.169	.532	-.030	.214	.120	.091	.093	.242
	p	.008	.000	.000	.000	.237	.000	.002	.014	.012	.000
	n	585	585	582	584	583	581	582	579	585	578
[5]Covid risk	r	.124	.092	.184	-.004	.721	.068	.020	.117	.295	-.008
	p	.001	.012	.000	.465	.000	.051	.316	.002	.000	.428
	n	592	592	589	590	590	585	589	586	592	585
[6]Practicality	r	.276	.434	.396	.303	.024	1.000	.263	.399	.362	.255
	p	.000	.000	.000	.000	.279	.000	.000	.000	.000	.000
	n	585	585	582	583	583	587	582	579	585	578
[7]	r	.220	.224	.336	.175	.030	.321	.710	.303	.206	.202

Bonding need	p	.000	.000	.000	.000	.233	.000	.000	.000	.000	.000
	n	590	590	587	588	588	587	587	584	590	583
[8]Boredom	r	.207	.235	.357	.155	.109	.399	.321	1.000	.264	.176
	p	.000	.000	.000	.000	.004	.000	.000	.000	.000	.000
	n	595	595	592	593	593	579	594	595	595	594
[9]Effective-ness Quarantine	r	.336	.266	.478	.145	.316	.352	.149	.228	.708	.112
	p	.000	.000	.000	.000	.000	.000	.000	.000	.000	.003
	n	591	591	588	589	589	584	588	585	591	584
[10]Financial disadvantage	r	.070	.130	.225	.243	-.041	.220	.191	.149	.097	.615
	p	.045	.001	.000	.000	.163	.000	.000	.000	.009	.000
	n	591	591	588	589	589	586	588	585	591	584

*p < .05, **p < .01, ***p < .001

Legend:

Quarantine-related psychosocial variables

[1]InfoCovid: feeling well informed about Covid-19

[2]Clear Protocol: clear communication about the quarantine protocol (purpose, lenths, rules, etc.)

[3]Social norms: Positive social norms of relevant others towards the quarantine (family, partner, fellow soldiers)

[4]Stigma: perceived stigma due tot he quarantine

[5]Covid risk: perceived risk by Covid-19 (self, family/partner, fellow sodliers, general)

[6]Practicality: being provided with everything needed during quarantine (daily nccessities, food, medical support)

[8]Boredom: quarantine-related boredom

[9]Effectiveness Quarantine: perceived benefit/effectiveness of quarantine (to protect self, family, fellow soldiers, vulnerable people, preventin deaths)

[10]Financial disadvantage: financial disadvantages caused by quarantining (additional costs for child-care, etc.)
