# Supplementary material for: Adherence and Psychosocial Well-Being During Pandemic-Associated Pre-deployment Quarantine
Source: Front Public Health. 2021 Dec 22;9:802180. doi: 10.3389/fpubh.2021.802180 (PMC8727777; doi:10.3389/fpubh.2021.802180)
Supplement: Supplementary file 12 [file Table_12.pdf]

Table 12: Relationship between quarantine adherence, mental health and quarantine-related psychosocial factors assessed at the beginning of pre-deployment quarantine
(All item values of the quarantine-related factors were z-standardized.)

		Adherence at the beginning of quarantine	Adherence at the end of quarantine	Mini_SCL at the beginning of quarantine	Mini_SCL at the end of quarantine
Adherence at the beginning of quarantine	r	1	.774	-.170	-.186
	p		.000	.000	.000
	n	597	592	590	585
Adherence at the end of quarantine	r	.774	1	-.155	-.303
	p	.000		.000	.000
	n	592	598	586	588
Mini_SCL at the beginning of quarantine	r	-.170	-.155	1	.417
	p	.000	.000		.000
	n	590	586	591	579
Mini_SCL at the end of quarantine	r	-.186	-.303	.417	1
	p	.000	.000	.000	
	n	585	588	579	591
[1]Info Covid	r	.276	.294	-.095	-.101
	p	.000	.000	.010	.007
	n	593	588	588	581
[2]Clear Protocol	r	.364	.376	-.140	-.192
	p	.000	.000	.000	.000
	n	593	588	588	581
[3]Social norms	r	.604	.599	-.181	-.155
	p	.000	.000	.000	.000

	n	592	587	587	580
[4]Stigma	r	.186	.128	-.137	-.124
	p	.000	.001	.000	.001
	n	587	582	582	576
[5]Covid risk	r	.299	.242	.071	.031
	p	.000	.000	.043	.228
	n	594	589	590	582
[6]Practicality	r	.348	.384	-.211	-.180
	p	.000	.000	.000	.000
	n	587	582	581	576
[7] Bonding need	r	.232	.294	-.273	-.227
	p	.000	.000	.000	.000
	n	592	587	586	581
[8]Boredom	r	.367	.467	-.050	-.194
	p	.000	.000	.115	.000
	n	589	592	583	588
[9]Effective-ness Quarantine	r	.473	.434	-.146	-.064
	p	.000	.000	.000	.063
	n	593	588	589	581
[10]Financial disadvantage	r	.096	.123	-.087	-.101
	p	.010	.001	.017	.007
	n	593	588	587	581

*p < .05, **p < .01, ***p < .001

Legend:

Quarantine-related psychosocial variables

[1]InfoCovid: feeling well informed about Covid-19

[2]Clear Protocol: clear communication about the quarantine protocol (purpose, lenths, rules, etc.)

[3]Social norms: Positive social norms of relevant others towards the quarantine (family, partner, fellow soldiers)

[4]Stigma: perceived stigma due tot he quarantine

[5]Covid risk: perceived risk by Covid-19 (self, family/partner, fellow sodliers, general)

[6]Practicality: being provided with everything needed during quarantine (daily nccessities, food, medical support)

[8]Boredom: quarantine-related boredom

[9]Effectiveness Quarantine: perceived benefit/effectiveness of quarantine (to protect self, family, fellow soldiers, vulnerable people, preventin deaths)

[10]Financial disadvantage: financial disadvantages caused by quarantining (additional costs for child-care, etc.)
