# Supplementary material for: Adherence and Psychosocial Well-Being During Pandemic-Associated Pre-deployment Quarantine
Source: Front Public Health. 2021 Dec 22;9:802180. doi: 10.3389/fpubh.2021.802180 (PMC8727777; doi:10.3389/fpubh.2021.802180)
Supplement: Supplementary file 13 [file Table_13.pdf]

Table 13: Relationship between mental health (Mini-SCL), quarantine adherence and quarantine-related psychosocialfactors assessed at the end of pre-deployment quarantine (All means are based on z-standardized item values.)

		Quarantine adherence (t2)	Mini-SCL (t2)
Adherence at the end of quarantine	r	1	-.303***
	p		.000
	n	598	588
Mini_SCL at the end of quarantine	r	-.303***	1
	p	.000	
	n	588	591
[1]Info Covid	r	.342***	-.144***
	p	.000	.000
	n	598	591
[2]Clear Protocol	r	.509***	-.299***
	p	.000	.000
	n	598	591
[3]Social norms	r	.659***	-.222***
	p	.000	.000
	n	595	588
[4]Stigma	r	.230***	-.286***
	p	.000	.000
	n	596	589
[5]Covid risk	r	.257***	.047
	p	.000	.127
	n	596	590
[6]Practicality	r	.384***	-.180***
	p	.000	.000
	n	582	576
[7] Bonding need	r	.307***	-.252***
	p	.000	.000
	n	595	590
[8]Boredom	r	.467***	-.194***
	p	.000	.000
	n	592	588
[9]Effective-ness Quarantine	r	.534***	-.139***
	p	.000	.000
	n	598	591
[10]Financial disadvantage	r	-.179***	.229***
	p	.000	.000
	n	591	587

*p < .05, **p < .01, ***p < .001

Legend:

t2= end of pre-deployment quarantine

Quarantine-related psychosocial variables

[1]InfoCovid: feeling well informed about Covid-19

[2]Clear Protocol: clear communication about the quarantine protocol (purpose, lenths, rules, etc.)

[3]Social norms: Positive social norms of relevant others towards the quarantine (family, partner, fellow soldiers)

[4]Stigma: perceived stigma due tot he quarantine

[5]Covid risk: perceived risk by Covid-19 (self, family/partner, fellow sodliers, general)

[6]Practicality: being provided with everything needed during quarantine (daily nccessities, food, medical support)

[8]Boredom: quarantine-related boredom

[9]Effectiveness Quarantine: perceived benefit/effectiveness of quarantine (to protect self, family, fellow soldiers, vulnerable people, preventin deaths)

[10]Financial disadvantage: financial disadvantages caused by quarantining (additional costs for child-care, etc.)
